# Cell Reprogramming Requires Silencing of a Core Subset of Polycomb Targets

**DOI:** 10.1371/journal.pgen.1003292

**Published:** 2013-02-28

**Authors:** Giulia Fragola, Pierre-Luc Germain, Pasquale Laise, Alessandro Cuomo, Alessandro Blasimme, Fridolin Gross, Elena Signaroldi, Gabriele Bucci, Cesar Sommer, Giancarlo Pruneri, Giovanni Mazzarol, Tiziana Bonaldi, Gustavo Mostoslavsky, Stefano Casola, Giuseppe Testa

**Affiliations:** 1 European Institute of Oncology, IFOM-IEO Campus, Milan, Italy; 2 IFOM Foundation, FIRC Institute of Molecular Oncology Foundation, IFOM-IEO Campus, Milan, Italy; 3 Boston University School of Medicine, Boston, Massachusetts, United States of America; 4 European Institute of Oncology, Milan, Italy; Friedrich Miescher Institute for Biomedical Research, Switzerland

## Abstract

Transcription factor (TF)–induced reprogramming of somatic cells into induced pluripotent stem cells (iPSC) is associated with genome-wide changes in chromatin modifications. Polycomb-mediated histone H3 lysine-27 trimethylation (H3K27me3) has been proposed as a defining mark that distinguishes the somatic from the iPSC epigenome. Here, we dissected the functional role of H3K27me3 in TF–induced reprogramming through the inactivation of the H3K27 methylase EZH2 at the onset of reprogramming. Our results demonstrate that surprisingly the establishment of functional iPSC proceeds despite global loss of H3K27me3. iPSC lacking EZH2 efficiently silenced the somatic transcriptome and differentiated into tissues derived from the three germ layers. Remarkably, the genome-wide analysis of H3K27me3 in *Ezh2* mutant iPSC cells revealed the retention of this mark on a highly selected group of Polycomb targets enriched for developmental regulators controlling the expression of lineage specific genes. Erasure of H3K27me3 from these targets led to a striking impairment in TF–induced reprogramming. These results indicate that PRC2-mediated H3K27 trimethylation is required on a highly selective core of Polycomb targets whose repression enables TF–dependent cell reprogramming.

## Author Summary

Multicellular organisms are composed of a variety of cell types. Over the last years we have learned that cell differentiation is fully reversible and that it takes few specific transcription factors (proteins that bind to DNA and regulate gene expression) to convert one cell type into another. The most dramatic example is the reprogramming of somatic cells into stem cells that reacquire the potential to give rise to all cell types of the body. This process entails the resetting of the gene expression program of the somatic cells necessary to acquire a pluripotent state but remains poorly understood. Here, we defined the role in cell reprogramming of the Polycomb axis, one of the key effectors of gene silencing that operates through a specific chemical modification of histones (the trimethylation of lysine-27 on histone H3) that is stably inherited from one cell generation to the next. Surprisingly, we uncovered a selective requirement for Polycomb silencing during somatic cell reprogramming. Upon inactivation of the essential Polycomb protein EZH2, histones trimethylated on lysine-27 were retained on a selected subset of physiological Polycomb target genes whose products control normal development, and erasure of the histone mark from these critical genes abolished reprogramming.

## Introduction

The realization that the expression of few transcription factors can reassign cell fate has been a paradigm-shifting insight for biology and medicine, from the pioneering conversion of fibroblasts into myoblasts [1], to the achievement of inducing pluripotency from adult somatic cells [2]. In particular, for the derivation of induced pluripotent stem cells (iPSC), the enticing medical opportunities during the past few years have focused most efforts on improving the efficiency and safety of transcription factor (TF)-induced reprogramming, and on defining the molecular and functional equivalence between iPSC and embryonic stem cells (ESC). Substantial progress has been made in the characterization of distinct stages of the reprogramming process (reviewed in [3]) as well as of its key features, such as the mesenchymal-to-epithelial transition [4], [5], and the importance of cell-cycle progression [6]–[11]. Yet, in terms of molecular understanding, we still lack a mechanistic insight of how the somatic gene expression program is silenced in order to enable the reacquisition of the pluripotent state. In particular, the functional relevance of defined chromatin modifications has only recently started to be explored [12]–[15], especially as far as those histone marks are concerned that orchestrate genome programming during development.

Genome-wide analyses have started to provide a detailed description of the chromatin changes that underlie TF-induced reprogramming to pluripotency. The very initial stages of the process entail the widespread acquisition of dimethylation of lysine-4 of histone H3 (H3K4me2) at promoters and enhancers of pluripotency genes that will be reactivated however only at later stages, indicating that this modification primes but does not directly trigger gene activation [16]. Conversely, analyses of established iPSC at the end of the reprogramming process revealed that changes in Polycomb-mediated trimethylation of lysine-27 of histone H3 (H3K27me3) represent a key feature that distinguishes the fibroblast from the iPSC epigenomes [17].

The Polycomb axis is organized around two families of protein complexes, Polycomb Repressive Complex 1 (PRC1) and Polycomb Repressive Complex 2 (PRC2) (reviewed in [18]). Both contain enzymatic activities that catalyze, respectively, monoubiquitylation of lysine-119 on histone H2A (H2AK119Ub) (carried out by RING1B and, to a lesser extent, RING1A) and di- and tri-methylation of lysine-27 of histone H3 (catalyzed by EZH2 and, to a lesser extent, EZH1) [18]. H3K27me3 constitutes a docking site for the chromodomain of CBX proteins, members of PRC1, leading to a cascade model for Polycomb action, in which PRC2 deposits H3K27me3 that then recruits PRC1 to enable H2AK119Ub [18]. Gene silencing by PRC2 is required for normal development and differentiation ([19] reviewed in [20]). In the mouse, inactivation of *Ezh2* or the essential non-enzymatic components of the complex, SUZ12 and EED, results in severe developmental failures *in vivo* and in varying degrees of impairment during ESC differentiation *in vitro* [21]–[27]. Thus, the finding that H3K27me3 constitutes the most significant chromatin mark that distinguishes somatic from iPSC epigenomes supports the notion that TF-induced reprogramming may represent the reverse trajectory of normal development and posits for this mark a key role in the repression of the somatic gene expression program. Furthermore, a hallmark feature of the ESC epigenome, fully shared with iPSC, is the presence of bivalent H3K4me3/H3K27me3 chromatin domains [28], [29]. These hold the promoters of key developmental regulator genes in a plastic state of minimal expression, poised for either full activation or definitive repression in a lineage-specific manner. Ablation of PRC2 components in ESC leads to the misexpression of many of these developmental targets [21], [22], indicating that bivalency is a critical feature of ESC pluripotency and predicting that its re-establishment is a necessary condition in iPSC reprogramming. Here we provide functional validation of the relevance of H3K27me3 in the reacquisition of pluripotency, through the conditional inactivation of *Ezh2* resulting in bulk depletion of H3K27me3 at the onset of TF-induced reprogramming. Our results demonstrate that, surprisingly, global H3K27me3 is dispensable for the reacquisition of the pluripotent state. Critically, however, we find that upon *Ezh2* inactivation, H3K27me3 is both retained and *de novo* acquired on a specific core of Polycomb targets involved in lineage determination and transcriptional regulation. This highly selective silencing of the somatic gene program is catalyzed by an alternative PRC2 and its inhibition impairs reprogramming, thus uncovering a critical but highly circumscribed role for H3K27me3 in TF-induced reprogramming.

## Results

### Derivation of iPSC from mouse embryonic fibroblasts upon *Ezh2* inactivation

**Figure 1:**
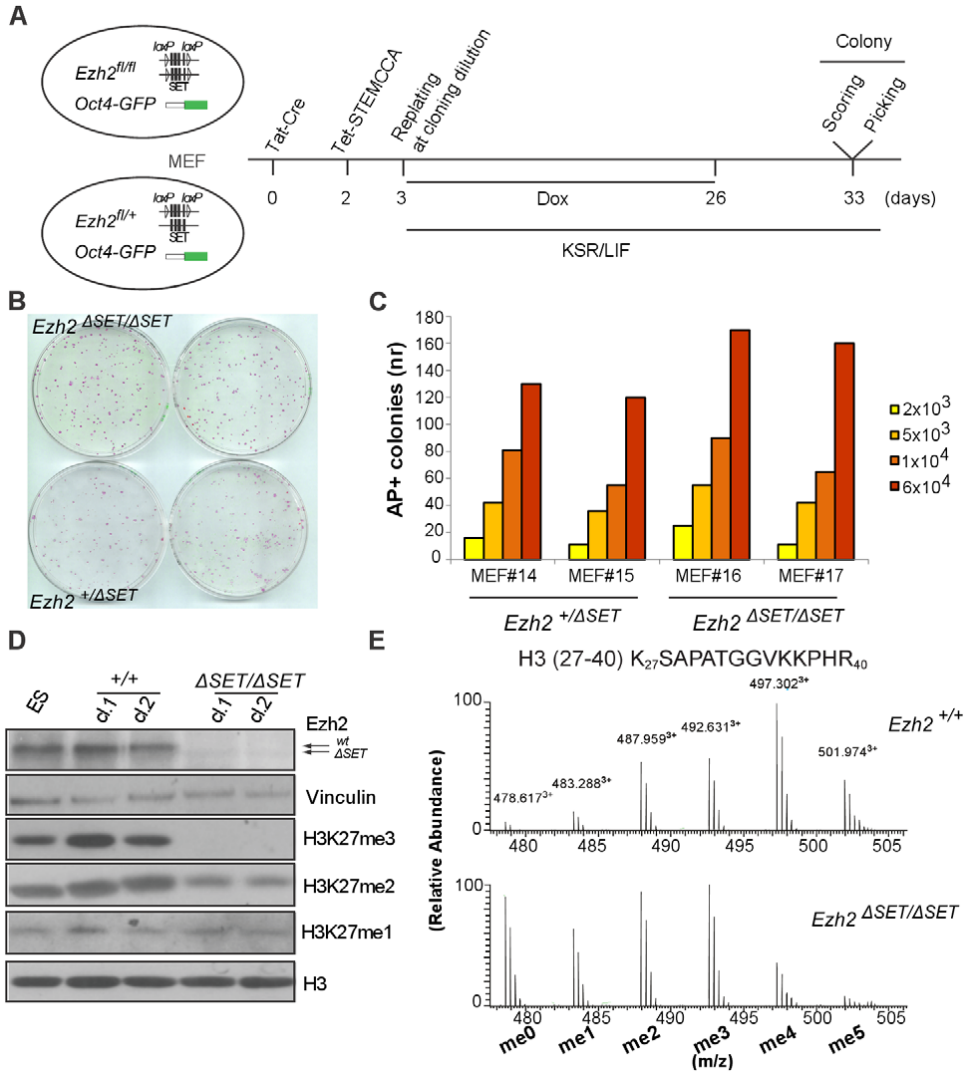
Derivation and biochemical analysis of iPSC upon conditional *Ezh2* inactivation. A. Diagram of the reprogramming protocol of MEF. B. Alkaline phosphatase staining of control (lower row) and mutant (upper row) primary iPSC colonies one week following doxycycline withdrawal. C. Number of AP-positive primary iPSC colonies obtained upon infection of, respectively, 2×10^3^, 5×10^3^, 1×10^4^ or 6×10^4^ MEF in two experiments performed with two biological replicates per genotype. D. EZH2, H3K27me1, H3K27me2 and H3K27me3 protein levels assessed by Western blot in two representative *Ezh2* control (*+/+*) and mutant *(ΔSET/ΔSET*) iPSC clones. Vinculin and Histone H3 were used as loading controls for, respectively, EZH2 and methylated forms of H3K27. E. Relative abundance in control (upper row) and mutant (lower row) iPSC clones of the six possible methylation isoforms of the Histone H3 peptide spanning amino acids 27–40, as determined by mass spectrometry.

In order to investigate the function of H3K27 trimethylation (H3K27me3) in TF-induced cell reprogramming, we derived embryonic fibroblasts (MEF) from mutant mice harboring a conditional *Ezh2* knock-out allele carrying loxP sites (*Ezh2^fl^*) flanking exons coding for the catalytic SET domain [30] (Figure 1 and Table 1). Experimental mice carried also an *Oct4-GFP* knock-in reporter gene [31] to monitor endogenous *Oct4* gene re-activation during reprogramming. We expressed *Oct4, Klf4, c-Myc* and *Sox2* reprogramming factors from a single doxycycline-regulated bicistronic lentiviral vector (STEMCCA), in which each cistron codes for a fusion of two factors which are then released through, respectively, F2A and E2A self-cleaving peptide signals [32]. As depicted in Figure 1A, our reprogramming protocol entailed: i) treatment of experimental (*Ezh2^f^* ^l/fl^; *Oct4-GFP*) and control (*Ezh2^+/fl^*; *Oct4-GFP)* MEF with cell-permeable TAT-Cre recombinase to inactivate *Ezh2*; ii) infection with STEMCCA and reverse tetracycline transactivator (*rtTA*) lentiviruses, followed by replating at clonal density and switch to a chemically-defined embryonic stem cell (ESC) medium (KSR) and administration of doxycycline for 23 days; iii) withdrawal of doxycycline to select cells that re-expressed the endogenous pluripotency factors; and iv) isolation, clonal propagation and molecular and functional characterization of individual iPSC clones. As controls, we used interchangeably iPSC from *Ezh2^fl/+^* MEF treated with TAT-Cre (referred to as *Ezh2^ΔSET/+^* iPSC) as well as iPSC from *Ezh2^+/+^*or *Ezh2^fl/fl^* MEF that were not exposed to Tat-cre (referred to as *Ezh2^+/+^* and *Ezh2^fl/fl^* iPSC). Upon verification of comparable efficiency in the infection with STEMCCA of control and mutant MEF (Figure S1A), we analyzed the efficiency of reprogramming following *Ezh2* inactivation by comparing the number of primary iPSC colonies assessed through staining with alkalyne phosphatase (AP) 7 days after doxycycline removal (Figure 1B). Starting from two different batches of MEF, we found no significant difference in the number of iPSC colonies generated from mutant (*Ezh2^ΔSET/ΔSET^*) and control (*Ezh2^ΔSET/+^*) fibroblasts (Figure 1B, 1C). As genome-wide chromatin analyses have postulated a critical role for H3K27me3 in the resetting of transcriptional programs during iPSC derivation [17], we tested whether this unexpected finding resulted from selective reprogramming of *Ezh2^fl/fl^* MEF that had escaped Cre-mediated recombination, thereby preserving H3K27me3-dependent reprogramming proficiency. We ruled out this possibility as the majority of iPSC clones analyzed carried the SET-deleted *Ezh2* allele regardless of the genotype (Figure S1B).

**Table 1:** Mass spectrometry analysis: H3K27me3 levels below the limit of detection in *Ezh2^ΔSET/ΔSET^* iPSCs.

		Mascot Score*		PTM Score**	
Peptide Forms	Modification Sites	*+/+*	*ΔSET/ΔSET*	*+/+*	*ΔSET/ΔSET*
me3	K27me3	48,72	ND	104,43	ND
me3	K27me1-K36me2	ND	38,55	ND	92,16
me3	K27me2-K36me1	52,3	20,02	117,35	32,14
me4	K27me2-K36me2	73,57	56,77	145,18	117,35
me4	K27me3-K36me1	47,61	ND	65,14	ND
me5	K27me3-K36me2	39,75	ND	92,16	ND

* Perkins et al 1999.

** Cox and Mann 2010.

Mascot and PTM scores attributed in control (*Ezh2^+/+^*) and mutant (*Ezh2^ΔSET/ΔSET^*) iPSC clones to each combination of amino acid modifications on the H3^27–40^ tri-, tetra- and penta-methylated peptide.

Representative iPSC clones of either genotype were analyzed by Western blot and found to be devoid of functional EZH2 and with undetectable H3K27me3 (Figure 1D). Levels of H3K27 dimethylation (H3K27me2) were also decreased in mutant iPSC clones, whereas H3K27 monomethylation (H3K27me1) remained unaltered (Figure 1D). Furthermore, global levels of the other major repressive histone modification, histone H3K9 trimethylation (H3K9me3), remained unaltered upon *Ezh2* inactivation (Figure S1C).

Global loss of H3K27me3 was confirmed by high performance liquid chromatography (HPLC) coupled to tandem mass spectrometry (MS/MS) analysis of the histone H3 fraction purified from control and mutant iPSC clones. Specifically, we determined the relative abundance of the peaks corresponding to the various combinations of modifications harbored by the H3 peptide spanning lysine-27 through arginine-40 (H3^27–40^). A significant change in the abundance and distribution of the various forms of methylated H3^27–40^ was observed (Figure 1E). We then carried out a detailed MS/MS fragmentation analysis to pinpoint methylation at specific residues, which indicated that H3K27me3 was undetectable in *Ezh2^ΔSET/ΔSET^* iPSC clones, confirming that changes in the relative abundance of methylated H3^27–40^are mainly attributed to loss of this modification (Table 1 and Figure S1E).

Finally, we measured by qRT-PCR the expression levels, in both mutant and control iPSC, of the exogenously provided reprogramming factors, to exclude that reprogramming in the absence of H3K27me3 had selected iPSC clones with a pronounced leakiness of the doxycycline-inducible transgenes, whose sustained expression enabled the maintenance of H3K27me3-depleted iPSC. As shown in Figure S1D, we excluded this possibility since EZH2-proficient and EZH2-deficient iPSC showed equivalent, minimal levels of transgene expression following doxycycline withdrawal.

### Self-renewal and pluripotency of *Ezh2^ΔSET/ΔSET^* iPSC

**Figure 2:**
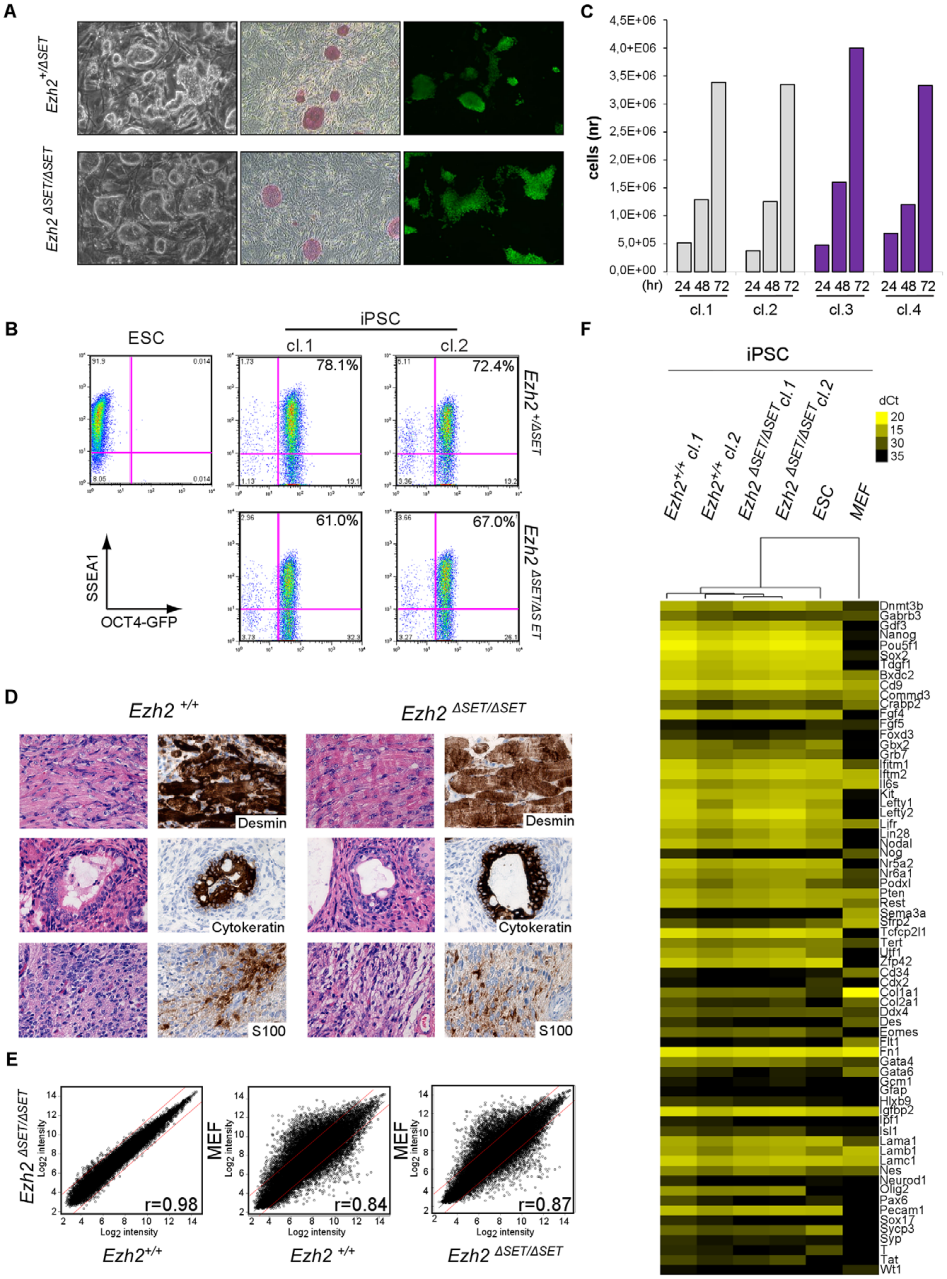
Characterization of pluripotency in iPSC clones reprogrammed upon *Ezh2* inactivation. A. Images of phase contrast (left), alkaline phosphatase staining (middle) and *Oct4*-driven GFP fluorescence (right) of representative control (*Ezh2^+/ΔSET^*, upper row) and mutant (*Ezh2^ΔSET/ΔSET^*, lower row) iPSC colonies. B. Flow cytometric analysis of SSEA-1 and endogenous OCT4 (OCT4-GFP) levels in representative control (*Ezh2^+/ΔSET^*, upper row) and mutant (*Ezh2^ΔSET/ΔSET^*, lower row) iPSC clones (cl.1 and cl.2). Embryonic stem cells (ESC) were used as positive control for SSEA1 expression (upper left). C. Growth curve of representative control (*Ezh^+/+^*, grey) and mutant (*Ezh2^ΔSET/ΔSET^*, purple) iPSC clones cultured in 2i/LIF medium for the indicated hours. Column height represents cell number. D. Hematoxylin & eosin staining and immunohistochemical analysis of representative sections of teratomas generated from either *Ezh2* control (*Ezh2^+/+^*) or mutant (*Ezh2^ΔSET/ΔSET^*) iPSC cells of two representative clones. Stainings for mesoderm (upper row), endoderm (middle row) and ectoderm (lower row) markers are displayed. Data displayed in A, B, C and D are representative of at least four independent experiments, using two iPSC clones per genotype. E. Scatter plots showing global gene expression correlation analyses between *Ezh2^+/+^* and *Ezh2^ΔSET/ΔSET^* iPSC (left panel), *Ezh2^+/+^* iPSCs and MEF (central panel) and *Ezh2^ΔSET/ΔSET^* iPSC and MEF (right panel). Correlation coefficients (r) reveal the degree of similarity for each comparison. Genes within red lines differ less than 1.5-fold. F. Heat map representation of expression levels of genes involved in pluripotency, stemness and differentiation in two control (*Ezh2^+/+^*, first and second column) and two mutant (*Ezh2^ΔSET/ΔSET^*, third and fourth column) iPSC clones. ESC (fifth column) and MEF (last column) were used for comparison. Colors range from yellow (low dCt, higher expression) to black (high dCt, lower expression). Hierarchical clustering of samples is also shown.

Having determined that *Ezh2* inactivation in MEF is compatible with TF-induced cell reprogramming, we performed a comprehensive functional characterization of representative control and mutant iPSC clones. *Ezh2^ΔSET/ΔSET^* and *Ezh2^ΔSET/+^* control MEF yielded iPSC colonies: i) with distinctive iPSC/ESC morphology (Figure 2A, left panel), ii) that stained positive for AP (Figure 2A, middle panel); and iii) that had reactivated the endogenous *Oct4* gene (as assessed by GFP fluorescence, Figure 2A, right panel). iPSC clones of either genotype had the same percentage of cells co-expressing the pluripotency markers OCT4 and SSEA1, as measured by flow cytometry (Figure 2B). Next we assessed control and mutant iPSC clones for the two cardinal features that define the pluripotent state: self-renewal and the ability to differentiate into cell types of the three germ layers.

Self-renewal is currently best assessed by growing ESC and iPSC under chemically defined conditions that entail the dual inhibition (2i) of mitogen activated protein kinase (MAPK) and glycogen synthase kinase 3 (GSK3) signaling, in the presence of leukemia inhibitory factor (LIF) (commonly referred to as 2i/LIF medium). These highly selective culture conditions were shown to capture the ground state of pluripotency exhibited by cells of the epiblast inner cell mass (ICM) [33]; importantly, they were also shown to selectively promote the full reprogramming of partially reprogrammed cells to the authentic pluripotent state [34]. As shown in Figure 2C, *Ezh2* mutant and control (*Ezh2^+/+^*) iPSC clones grew readily in 2i/LIF and have been cultivated for over 20 passages. Finally, we tested the ability of *Ezh2*-proficient (*Ezh2^+/+^*) and mutant (*Ezh2^ΔSET/ΔSET^*) iPSC to differentiate along the three embryonic lineages through the formation of teratomas. iPSC clones of either genotype (2 clones for each genotype) injected into NOD/SCID *Il2rγc^−/−^*immunodeficient mice gave rise within three to four weeks to teratomas harboring terminally differentiated cell types derived from the three germ layers. Differentiation was assessed combining hematoxylin/eosin with immunohistochemical stainings for lineage-specific markers (desmin for the mesodermal, S-100 for the neurectodermal and cytokeratin for the endodermal and ectodermal lineages, respectively) (Figure 2D).

Next, we asked to which extent the transcriptome of *Ezh2* mutant iPSC had been reset correctly during reprogramming. As shown in Figure 2E and Figure S2, a stringent analysis of 4 independent control and mutant iPSC clones (*t*-test, FDR <0.05) revealed that transcriptomes were indistinguishable from each other and equally divergent from MEF-specific ones. We validated this result through qRT-PCR on a panel of well-established genes associated with pluripotency, stemness and lineage specification (n. 7, 32 and 50 genes, respectively). Figure 2F shows a heat map representation of the expression levels (expression data of all genes are provided in Table S1), with iPSC clones of both genotypes clustering together with ESC and sharply distinguished from MEF.

### Bulk H3K27me3 is dispensable from the onset of reprogramming

**Figure 3:**
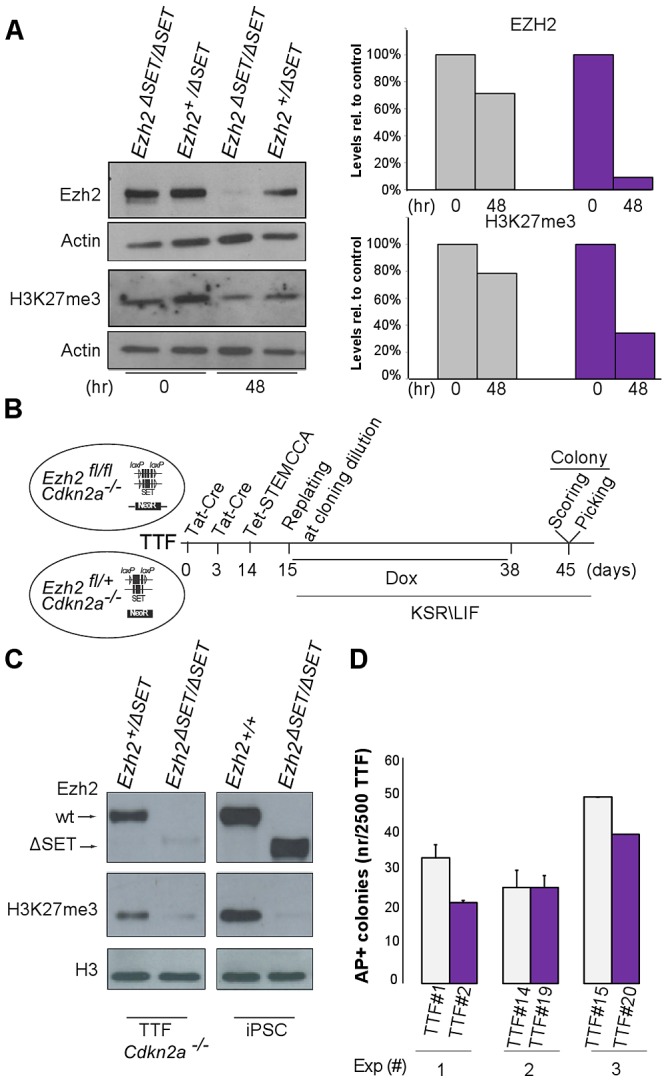
Establishment of iPSC clones upon genome-wide erasure of H3K27me3 at the onset of reprogramming. A. Western blot analysis of EZH2 and H3K27me3 protein levels respectively at onset or 48 hr after reprogramming in *Ezh2^+/ΔSET^* and *Ezh2^ΔSET/ΔSET^* MEFs. Data are representative of two experiments. Quantification of protein levels at the indicated time points is shown in the right panel (controls in grey, mutants in purple). B. Strategy to induce reprogramming of tail tip fibroblasts (TTFs) lacking H3K27me3 at the onset of reprogramming. C. Western blot analysis of EZH2 and H3K27me3 protein levels in *Cdkn2a^−/−^* TTF carrying either one (+/*Δ*SET) or both (*Δ*SET/*Δ*SET) *Ezh2* mutant alleles. As comparison, representative *Ezh2*-proficient (+/+) and -deficient (*Δ*SET/*Δ*SET) iPSC clones were analyzed. The band corresponding to H3K27me3 in mutant TTF has the same intensity of that from a mutant iPSC clone for which mass-spectrometry did not detect the presence of H3K27me3. D. Quantification of AP-positive primary iPSC colonies obtained from infection of 2.5×10^3^ *Cdkn2a^−/−^* TTF, respectively proficient (grey bar) or deficient (purple) for *Ezh2*, assessed in three independent experiments.

TAT-Cre-mediated inactivation of *Ezh2* resulted in a threefold decrease in global H3K27me3 levels by the onset of the reprogramming process (Figure 3A). We therefore asked whether the unexpected possibility to reprogram despite inactivation of *Ezh2* was due to residual H3K27me3 that could have still ensured, during the very first days of reprogramming, a sufficient degree of repression of lineage specific genes. To this end, we aimed at erasing the H3K27me3 mark completely by dilution, through serial passage of TAT-Cre treated MEF before the start of reprogramming. In MEF, however, PRC2 is a direct repressor of *Cdkn2a*, a locus encoding three key cell cycle regulators (p16, p19 and p15) whose activation promotes senescence [35]. Importantly, expression of this locus, and in particular of *p19/Arf*, was shown to hinder iPSC reprogramming [9], Thus, to prevent senescence driven by *Cdkn2a* de-repression following *Ezh2* inactivation (Figure S3A), we resorted to compound primary tail tip fibroblasts (TTF) harboring both the conditional *Ezh2* allele and the *Ink4/Arf* knock-out allele [36]. We subjected TTF to two sequential rounds of TAT-Cre transduction and passaged them 5 times before infection with STEMCCA and doxycycline administration (Figure 3B). Following confirmation that H3K27me3 was undetectable by Western blot on the day of infection with the reprogramming lentivirus (Figure 3C), and that efficiency of infection was equivalent for control and mutant TTF (Figure S3B), we went on to measure the efficiency of TF-dependent reprogramming under these most stringent conditions. As shown in Figure 3D, AP staining revealed that TTF starting off with undetectable H3K27me3 and controls yielded iPSC colonies with similar efficiency. This indicates that bulk levels of H3K27me3 are not required to prime silencing during the first days of reprogramming.

### *Ezh2* depleted iPSC retain H3K27me3 on selected Polycomb targets

**Figure 4:**
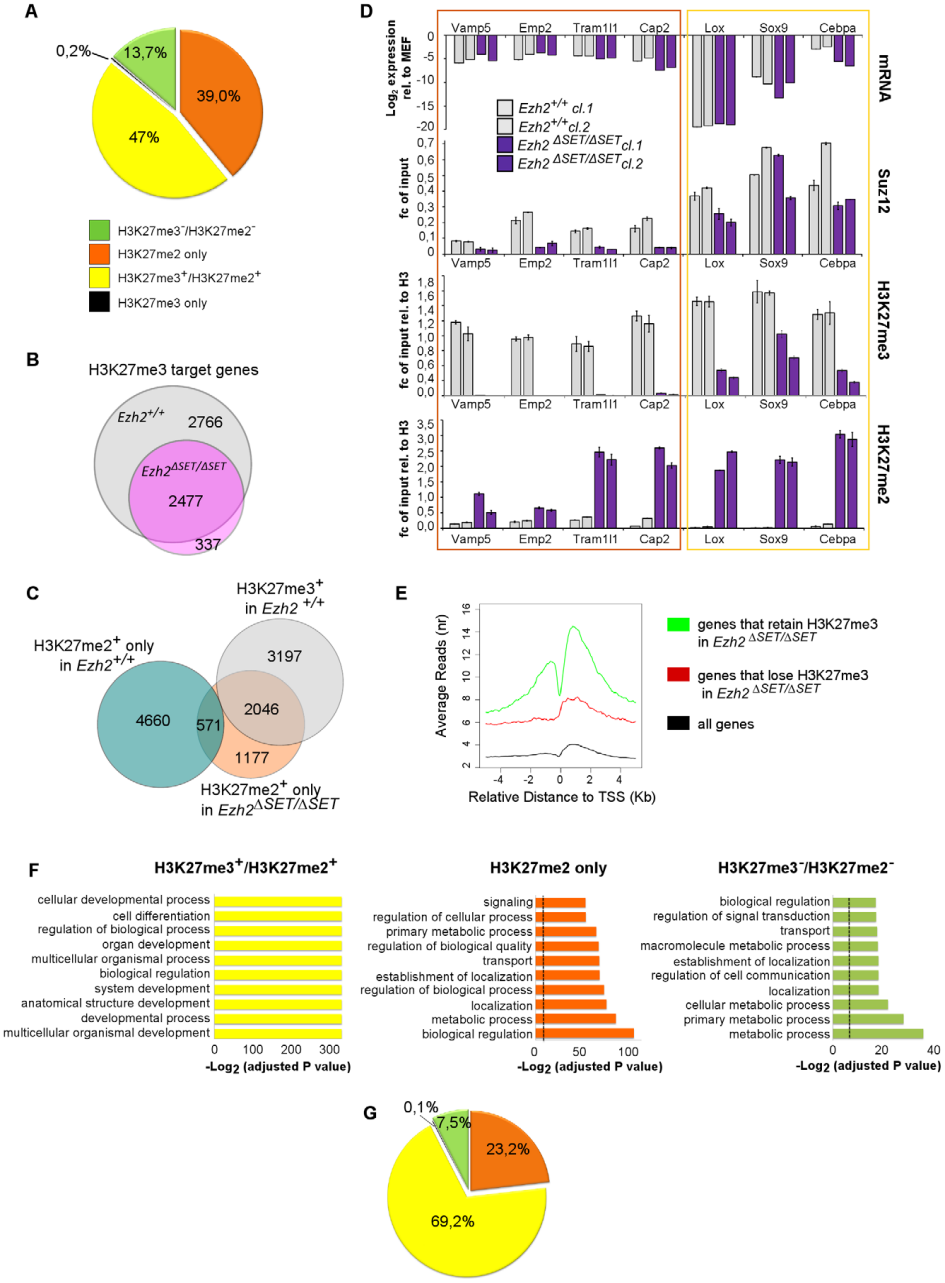
Genome-wide distribution of H3K27me3 in *Ezh2^ΔSET/ΔSET^* iPSC revealed through ChIP–seq. A. Pie chart showing partition of Polycomb targets based on H3K27 methylation status in *Ezh2^ΔSET/ΔSET^* iPSCs. B. Venn diagram displaying overlap between H3K27me3^+^ genes in *Ezh2* ^+/+^ (grey) and *Ezh2^ΔSET/ΔSET^* (purple) iPSC. C. Venn diagram showing overlap between H3K27me3^+^ genes in *Ezh2* ^+/+^ iPSC (grey), H3K27me2^+^/H3K27me3^−^ genes in *Ezh2^ΔSET/ΔSET^* iPSC (orange) and H3K27me2^+^/H3K27me3^−^ in *Ezh2* ^+/+^ iPSC (light blue). D. Analysis of transcript levels measured by qRT-PCR, and status of SUZ12 binding, H3K27me2 and H3K27me1 enrichment revealed by ChIP q-PCR at promoters of 7 genes overexpressed in MEF relative to iPSC and representative of groups of genes marked by either H3K27me2^+^/H3K27me3^+^ or H3K27me2^+^/H3K27me3^−^ in *Ezh2^ΔSET/ΔSET^* iPSC. For all analyses, two *Ezh2* control (+/+; grey) iPSC clones were compared to two mutant (*Δ*SET/*Δ*SET; purple) counterparts. Levels of expression are shown as ddCt (log_2_ scale) relative to MEF. Status of a particular histone modification (±SEM) is represented as fold change of enrichment relative to input, after normalization for H3 density within the same amplicon. SUZ12 enrichment at promoters of indicated genes was determined comparing it to that of an unrelated IgG. Error bars refer to qPCR triplicates. E. Average reads distribution of H3K27me3 around the TSS in *Ezh2* ^+/+^ iPSC. Genes were divided in two classes based on H3K27me3 status in *Ezh2^ΔSET/ΔSET^* iPSC (H3K27me3^+^ genes in green, H3K27me3^−^ genes in red) and compared to all genes (black) F. Gene ontology analysis of genes belonging to the three main groups based on H3K27 methylation status in *Ezh2^ΔSET/ΔSET^* iPSC depicted in panel A. Bars represent *P*-values in –Log_2_ scale of the corresponding biological processes. Dashed lines identify the significance threshold. G. Pie chart of H3K27 methylation status in *Ezh2^ΔSET/ΔSET^* iPSC of MEF specific genes marked by H3K27me3 in *Ezh2* ^+/+^ iPSC. Color code is shown in panel A.

The finding that bulk H3K27me3 was apparently dispensable for reprogramming even when erased at the onset of the process was at odds with its purported role as the critical mark that distinguishes MEF from iPSC epigenomes as well as, more broadly, with its pivotal role in the maintenance of gene repression through embryogenesis and adulthood [17]. We therefore asked whether, upon *Ezh2* inactivation, residual levels of H3K27me3 below the threshold of Western blot and mass spectrometry sensitivity, could still be deposited on selected targets. To address this point we performed chromatin immunoprecipitation coupled to high-throughput sequencing (ChIP-seq). We generated ChIP-seq profiles from two independent control and mutant iPSC clones for both H3K27me3 and H3K27me2 with highly specific monoclonal antibodies. Consistently with our prediction, the higher sensitivity of ChIP-seq did reveal the presence of residual H3K27me3 in mutant iPSC clones. Specifically, the mark was retained on 2477 genes (with an enriched region overlapping a +/−5kb region interval around the transcriptional start site, TSS), comprising close to half of all H3K27me3 targets retrieved from wild type iPSC. Mutant clones showed a preferential retention of H3K27me3 proximal to the TSS of target genes. In comparison to the full complement of PRC2 targets in control iPSC, mutant clones displayed a clear tripartition in the genome-wide distribution of H3K27me3 and H3K27me2 marks (Figure 4A). 47% of genes retained both H3K27me3 and H3K27me2, 39% of genes were marked only by H3K27me2 and 13,7% of genes lost both marks. Importantly, the complement of genes enriched for H3K27me3 in mutant iPSC clones was almost entirely comprised within the group of H3K27me3 targets found in control iPSC cells, thus excluding a significant redistribution of the mark to new targets in cells reprogrammed in the absence of functional *Ezh2* (Figure 4B). Furthermore, we found only a small overlap in the distribution of H3K27me2-only targets between control and mutant iPSC clones. Instead, the complement of genes marked only by H3K27me2 in mutant iPSC clones was to a good extent comprised within the subset of genes that are H3K27 trimethylated in control iPSC (Figure 4C). Thus, we conclude that during reprogramming in the absence of *Ezh2*, i) H3K27me3 is selectively retained on a subset of the targets that are normally H3K27 trimethylated in iPSC, where it coexists with H3K27me2; ii) H3K27me2 is lost at targets that are normally carrying only this mark in iPSC; and iii) H3K27me2 is retained in 86% of the targets that are normally H3K27 trimethylated in iPSC, coexisting, in half of these, with residual H3K27me3. We validated these findings through individual ChIP-qPCR on genes selected among those that were downregulated in the MEF to iPSC transition (Figure 4D and Table S2). We confirmed the sharp distinction between a group of genes that retained both H3K27me3 and H3K27me2 and those that only retained H3K27me2, irrespective of the level of transcriptional repression that was equivalent for the two groups between control and mutant iPSC (Figure 4D and Table S2). Interestingly, we found a stronger enrichment for PRC2 on the genes that selectively retained the H3K27me3 mark, likely reflecting its ability to act as docking site for the EED subunit of PRC2.

In the absence of EZH2, EZH1 is the only other known enzymatic component of PRC2, where its enzymatic activity is however twenty fold weaker than that of EZH2 [37]. Our findings support therefore a model in which, in the presence of EZH1-only PRC2 complexes, genes that are normally more enriched for PRC2 will be more likely to accumulate H3K27me3 because they will have a higher chance of having the weak activity of EZH1 result in productive trimethylation. In turn this will further enhance PRC2 recruitment, establishing a feed-forward loop that guarantees on selected targets residual levels of H3K27me3. We tested this hypothesis by analyzing, in control iPSC, the average enrichment for H3K27me3 in genes that, in mutant iPSC, respectively retain or lose this mark. As shown in Figure 4E and Figure S4B, genes that retain H3K27me3 in mutant iPSC showed indeed a highly significant stronger enrichment for this mark in control iPSC cells, both in terms of maximum height and average read density (p-value<2.2e-16, two tailed t-Test). In almost all cases however, targets that retain H3K27me3 in mutant iPSC also feature H3K27me2 (contrary to what happens in control iPSC), indicating that EZH1 does not reach H3K27 trimethylation: 1) in all cells of the population, 2) on both alleles of the same cell, or 3) on both histone H3 tails of the same nucleosome. The non-randomness of H3K27me3 marking in mutant iPSC was confirmed by the virtually identical genome-wide distribution of H3K27me3 and H3K27me2 in two independent iPSC mutant clones (Figure S4D), as well as by the striking similarity between the distributions of, respectively, H3K27me3 in control and H3K27me2 in mutant iPSC (Figure S4C).

### Targets of H3K27me3 in *Ezh2* mutant iPSC are enriched for transcriptional regulators and developmental determinants

Finally, we asked whether the H3K27me tripartition of the mutant iPSC epigenome identified functionally relevant classes of genes. To this end we analyzed the three groups of PRC2 targets differentially methylated in mutant iPSC (H3K27me3^+^/H3K27me2^+^; H3K27me3^−^/H3K27me2^+^; and H3K27me3^−^/H3K27me2^−^) in terms of the Gene Ontology (GO) of their members (Figure 4F). Remarkably, the subset of genes that retained H3K27me3 in mutant iPSC showed a distinct GO profile when compared to the other two classes, with a clear enrichment for categories linked to development, cellular differentiation and transcriptional regulation. This unique signature was confirmed when the GO enrichment in H3K27me3-retaining genes was probed against the sole complement of all Polycomb targets, further underscoring the functional partition of the Polycomb epigenome. The H3K27me3^−^/H3K27me2^+^ and H3K27me3^−^/H3K27me2^−^ subsets were instead strongly enriched for genes involved in metabolic homeostasis and cellular transport.

Remarkably, while genes that retained H3K27me3 in mutant iPSC comprised 47% of the physiological PRC2 epigenome, they were disproportionately enriched for genes preferentially expressed in MEF versus iPSC (comprising 69,2% of the total, Figure 4G). This suggested that retention of H3K27me3 in *Ezh2* mutant iPSC occurred on selected targets to enable silencing of the MEF-specific gene expression program. Specifically, we found 550 genes, among those that retained H3K27me3 in mutant iPSC, that were downregulated in the MEF to iPSC transition (Figure S5A). Among these, 175 acquire H3K27me3 *de novo* in the MEF to iPSC transition and are enriched in GO categories related to transcriptional regulation (Figure S5B). We hypothesized the presence within this group of one or more MEF-specific master regulators that were silenced during reprogramming through H3K27me3. We thus performed a master regulator analysis (MRA) to identify the TFs whose targets (predicted on the basis of their consensus binding sites) are over-represented in the differentially expressed genes (DEGs) between MEF and iPSC. To this end we applied the transcription factor binding site over-representation analysis algorithm [38]. This analysis uncovered four TFs, *Egr1, Ets1, Prxx1, Prxx2*, whose binding sites were significantly over-represented (FDR<5%) among the 175 DEGs that acquire *de novo* H3K27me3 in the transition from MEF to iPSC, and are predicted to control the bulk (84%) of the up-regulated genes in MEF with respect to iPSC (Figure S5C). Notably, expression of *Egr1* and *Ets* transcript levels showed a strong reduction within the first week of reprogramming, corroborating their role in the silencing of the MEF-specific program (Figure S5D).

### PRC2 catalyzes residual H3K27 trimethylation in *Ezh2* mutant iPSC

**Figure 5:**
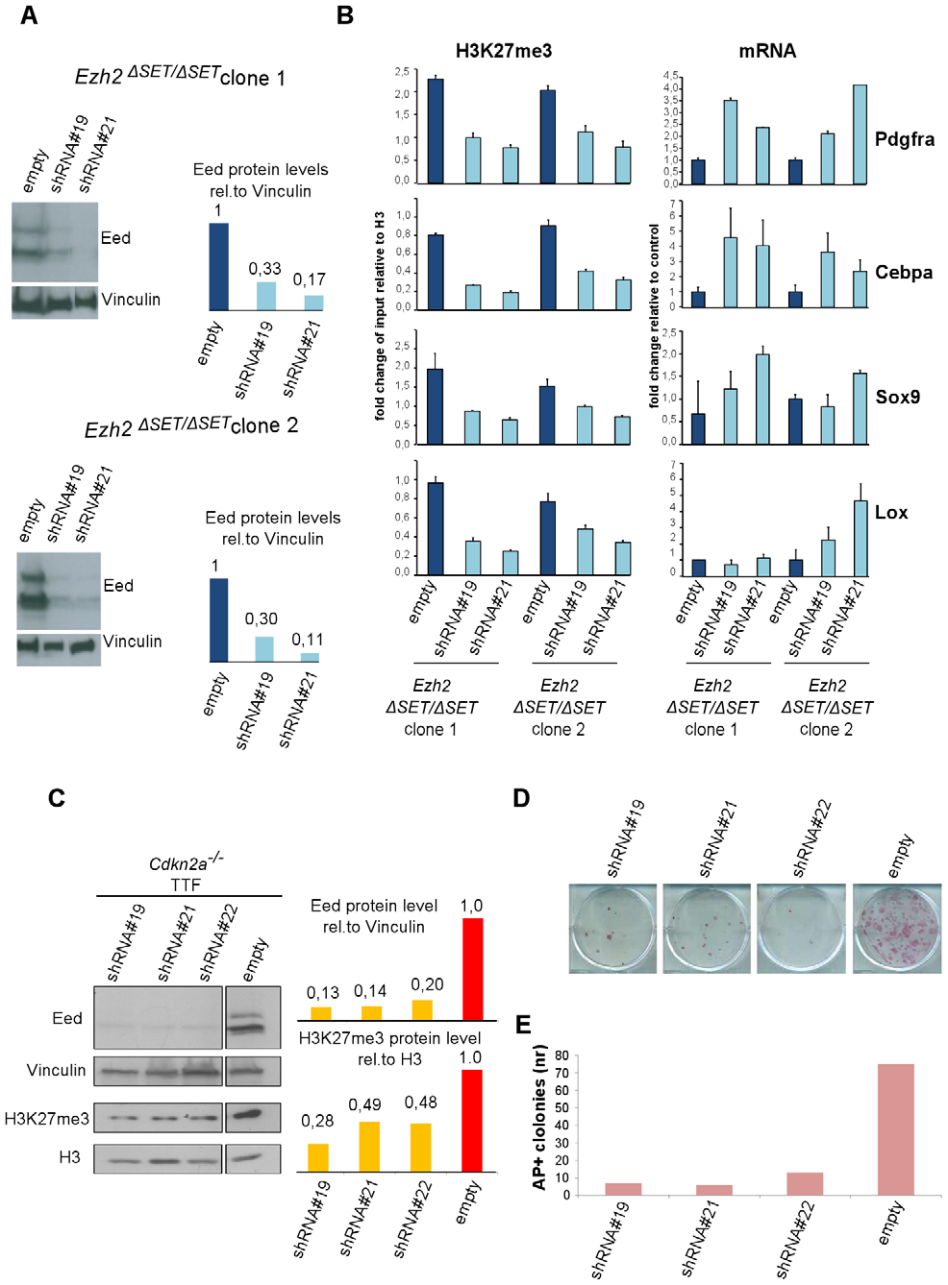
Effect of PRC2 inactivation on established *Ezh2^ΔSET/ΔSET^* iPSC clones and TF–induced reprogramming. A. Western blot analysis of EED protein levels in two *Ezh2^ΔSET/ΔSET^* iPSC clones infected with control virus (empty) or lentiviruses expressing independent short hairpin (sh) RNAs targeting *Eed* (#19 and #21)(left). Quantification of EED protein levels in infected cells after normalization based on Vinculin levels (right). B. H3K27me3 status (left panel) and expression levels (right panel) measured respectively by ChIP-qPCR and qRT-PCR, of 4 representative genes up regulated in MEF relative to iPSC, in two *Ezh2^ΔSET/ΔSET^* iPSC clones. *Ezh2*-mutant iPSC were infected with viruses expressing two independent hairpins for *Eed* or with a control virus. Status of H3K27me3 (±SEM) is represented as enrichment relative to input, after normalization for H3 density within the same amplicon. Expression levels are shown as fold change relative to iPSC infected with the empty vector. Error bars refer to qPCR triplicates. C. Western blot analysis of EED and H3K27me3 protein levels at day-6 of puromycin selection on *Cdkn2a^−/−^ Ezh2*-proficient TTF expressing three independent *Eed* hairpins (lines 1, 2 and 3) or infected with a control virus (line 4). Quantification of protein levels relative to Vinculin or Histone H3 are shown in the right panel. D. AP staining of primary iPSC colonies obtained upon reprogramming of 1×10^3^ (upper panel) *Cdkn2a^−/−^ Ezh2*-proficient TTF expressing three independent *Eed* hairpins (lines 1, 2 and 3) or infected with an empty lentiviral vector (line 4) used as control. E. Quantification of AP^+^ iPSC colonies. Column height represents number of AP^+^ iPSC colonies obtained from 1×10^3^ TTF expressing either one of the three independent *Eed* hairpins (lines 1, 2 and 3) or infected with an empty lentivirus vector (line 4) as control. Data are representative of two independent experiments performed using three different shRNAs.

Previous reports have highlighted partial redundancy between *Ezh2* and *Ezh1* in several cell types [27], [39]. To determine whether an alternative PRC2 complex is responsible for H3K27me3 of target genes also in *Ezh2*-mutant iPSC, we knocked down the essential PRC2 component EED in *Ezh2*-mutant iPSC. Infection of two *Ezh2*-mutant iPSC clones with lentiviruses expressing two independent shRNAs against *Eed* promoted a substantial reduction of EED protein levels (Figure 5A). *Ezh2*-mutant iPSC stably interfered for *Eed* (or infected with a control virus) were subjected to ChIP-qPCR assays on a representative set of target genes retaining the H3K27me3 mark in mutant iPSC. As shown in Figure 5B, levels of H3K27me3 decreased substantially in two independent *Ezh2*-mutant iPSC clones upon *Eed* knock-down. Importantly, loss of H3K27me3 led to the reactivation of target gene expression (Figure 5B, right panel). These results indicate that an alternative EZH1-containing PRC2 complex deposits H3K27me3 on a selected subset of Polycomb targets during reprogramming of *Ezh2*-mutant iPSC, to promote stable gene repression.

Given the existence of an alternative PRC2 complex that functions in *Ezh2* mutant iPSC, we determined the effect of *Eed* knock-down on TTF reprogramming. Specifically, *Ezh2*-proficient *Cdkn2a^−/−^* TTFs were infected first with a lentivirus expressing a shRNA against *Eed*, and few days later with the STEMCCA reprogramming virus (Figure S6A). As control, TTF cells were infected first with a control virus followed by STEMCCA. Quantification of iPSC colonies by AP staining performed two weeks after the beginning of doxycycline treatment revealed an over 80% reduction in reprogramming efficiency upon *Eed* inactivation (Figure 5E), indicating that PRC2-mediated H3K27me3 deposition on a selected subset of Polycomb targets is necessary for correct fibroblast reprogramming.

## Discussion

Trimethylation on lysine-27 of histone H3 by Polycomb proteins has been characterized as a critical mechanism that ensures the cell-type specific establishment and maintenance of gene silencing in multicellular organisms [19], [20], [40], [41]. From ESC to tissue-specific stem cells, this chromatin mark has been associated to the timed repression of developmental regulator genes, which underlies in turn the physiological unfolding of cell fate transitions [19]–[22], [42]–[44]. Consistent with its role in the physiology of genome programming during development, the Polycomb axis has been hypothesized as a key player in the reprogramming of somatic cells epigenomes. In particular TF-induced reprogramming, through its relatively high efficiency coupled with the stability of the reprogrammed state and the ability to study defined stages of the process, enabled the first insight into the chromatin changes that underlie cell fate reassignment on a genome-wide scale. Among 16,500 promoters that were analyzed by ChIP-on-chip, roughly 1000 differed sharply in the H3K4me3 and H3K27me3 status when MEF were compared to ESC [17]. Nearly all of these signature genes displayed H3K4me3 and H3K27me3 patterns that were identical between ESC and iPSC, thus providing the first evidence that the transition from MEF to iPSC recapitulated, in addition to the transcriptome, to a large extent also the epigenome of ESC. Importantly, however, a comparison of methylation maps between MEF, ESC and iPSC revealed that H3K4me3 changed considerably less between the three cell types than H3K27me3, whose distribution was instead remarkably different between MEF, on the one hand, and ESC and iPSC on the other. This observation suggested that changes in H3K27me3 were more relevant to reprogramming than those in H3K4me3, and led to posit for the Polycomb pathway a central role in the conversion of MEF into iPSC [17]. A similar approach was used to investigate the earliest stages of the reprogramming process, uncovering the acquisition of H3K4 dimethylation (H3K4me2) at several pluripotency loci and developmental regulators that precedes their transcriptional activation [16]. More recently the inactivation of the H3K27 demethylase UTX revealed the essential role of H3K27 demethylation in TF-induced reprogramming, through the reactivation of a handful of pluripotency genes during the initial stages of the process [13]. Similarly, Polycomb affected the ability of ESC to reassign fate in cell fusion-based short term reprogramming [45]. Thus, while these data reveal the importance, in the early phase of reprogramming, of the reactivation of the pluripotency network through widespread H3K4me2 acquisition and loss of H3K27 methylation, the relevance of H3K27 trimethylase activity in silencing somatic gene expression remains still ill defined.

Here we provide functional validation for the role of H3K27me3 in TF-induced reprogramming. Our findings reveal that functional inactivation of EZH2, the main catalyst of H3K27 trimethylation within PRC2, is surprisingly compatible with TF-cell reprogramming. In its absence, reprogramming proceeds through the deposition of H3K27me3 by the alternative EZH1-PRC2, on a highly selected core of Polycomb targets controlling lineage determination and transcriptional regulation. Moreover, our data show that correct reprogramming requires an exquisitely low amount of H3K27me3 below the limit of detection of mass spectrometry. Bioinformatics analysis of ChIP-seq data revealed that H3K27me3 is retained in *Ezh2* mutant iPSC on about 2500 genes, representing close to half of all PRC2 targets in iPSC and ESC. These include 2190 genes that are already marked by H3K27me3 in MEF, with the remaining ones acquiring the modification during the MEF to iPSC transition, thus indicating that residual PRC2 is able through cell division to both propagate the mark at pre-existing loci and redistribute it to new targets. The correct reprogramming guaranteed by selective retention of H3K27me3 was also consistent with the normal differentiation of *Ezh2*-mutant iPSC in teratoma assays, defining the core subset of functionally relevant Polycomb targets. Similar results were obtained also when *Ezh2* was inactivated in established iPSC (data not shown). These data exclude a functional difference between iPSC reprogrammed *ab initio* in the absence of *Ezh2* or acutely depleted of the enzyme following reacquisition of pluripotency. In turn, this is consistent with the observation that H3K7me3 is partially retained upon *Ezh2* inactivation.

Importantly, fully independent mutant iPSC clones were indistinguishable in their patterns of residual H3K27me3, indicating that retention of the mark either follows an instructive mechanism or, if stochastic, is subjected to a strong selection pressure that limits the range of residual targets. The former possibility appears more likely on the basis of two related observations: first, that residual H3K27me3 targets in mutant iPSC are preferentially associated to CpG islands, and second, that these residual H3K27me3 targets also correspond to genes with higher levels of H3K27me3 in normal iPSC. Combined, these evidences suggest that the basic mode of PRC2 recruitment is conserved in *Ezh2*-mutant cells, allowing prevalent targets to recruit enough mutant PRC2 for EZH1 to catalyze productive H3K27 trimethylation.

In mutant iPSC, further inhibition of PRC2 through knock-down of its critical subunit EED resulted in loss of H3K27me3 and re-expression of developmental regulator genes, indicating that maintenance and/or *de novo* acquisition of H3K27me3 is critical to ensure silencing of the MEF-specific gene expression program. Consistently, *Eed* knock-down in MEF prevented reprogramming. This result highlights the essential contribution of H3K27me3 to the repression of developmental regulator genes that enables successful cell fate reassignment.

## Materials and Methods

### Mice

*Ezh2^fl^, OCT4-GFP* and *Cdkn2a^−/−^* mice have been previously described [30], [31], [36]. Compound mutants were generated intercrossing individual strains. Primers used for genotyping are listed in Table S5.

### Derivation of primary MEF and TTF

MEF were produced from E13.5 compound mutants following standard procedures. Briefly, embryos were harvested from pregnant females and MEF were isolated by enzymatic dissociation of tissues. Cells were cultured in MEF medium (DMEM high-glucose, 10% fetal calf serum, 2mM L-glutamine, 50 units/ml penicillin, 50 µg/ml streptomycin) at 37°C, 5% CO2 for up to passage-3 before TAT-Cre transduction and/or infection with reprogramming viruses. For the preparation of adult tail tip fibroblasts (TTF), 0.5 cm tail tips from 8 to 16-weeks old compound mutants were biopsied aseptically, subjected to enzymatic dissociation and cultured in MEF medium at 37°C, 5% CO2 for one week.

### Lentivirus production

Lentiviral constructs coding for doxycycline-inducible STEMCCA, reverse tetracycline transactivator (*rtTA*) and the Zs-green reporter gene have been previously described [32]. Production of lentiviral particles was performed as previously described [32]. Briefly, plasmids expressing viral proteins GAG, POL, REV, TAT, and the vesicular stomatitis virus envelope glycoprotein (VSV-G) were co-transfected with either STEMCCA or *rtTA* vectors into semi-confluent 293T cells by calcium phosphate precipitation in the presence of 25 µM chloroquine. Supernatant of transfected cells were collected every 12 hours during 2 consecutive days and concentrated by centrifugation. Viral particles were resuspended in MEF medium and either used freshly for infection or frozen at −80°C.

### Infection of MEF and derivation of *Oct4-GFP*; *Ezh2^fl^* iPSC

MEF at passage 1 to 3 carrying either one (control) or both (mutant) *Ezh2* floxed alleles were seeded at a density of 2×10^5^ cells/well in a 6-well tissue culture dish. 24hr later, MEF were transduced in a 1∶1 PBS/DMEM/GlutaMAX (Invitrogen) solution with 50 µg/ml of recombinant TAT-Cre recombinase [46]. 16 hour after transduction, medium was replaced, and MEF were infected with the STEMCCA and *rtTA* lentiviruses. 24 hours following infection with STEMCCA and *rtTA*, MEF were harvested and seeded at clonal density on a mytomycin-treated MEF feeder layer. One day after, MEF medium was replaced with ESC medium (high glucose DMEM, 15% knockout serum replacement-Invitrogen-, 2 mM L-glutamine, 50 units/ml penicillin, 50 µg/ml streptomycin, 0,1 mM non-essential amino acids, 1/500 home-made leukaemia inhibitory factor, 0,1 mM 2-β-mercaptoethanol) supplemented with 1 µg/ml doxycycline to induce expression of the reprogramming factors. Doxycycline treatment was replaced every other day for 23 days. Four days after doxycycline withdrawal, iPSC colonies were stained for alkaline phosphatase (AP), counted to assess the efficiency of reprogramming or picked and expanded on a mitomycin-treated MEF feeder layer to establish individual iPSC clones. To measure the efficiency of infection, an aliquot of MEF of each genotype was infected in parallel with a combination of STEMCCA, *rtTA* and a lentivirus expressing ZsGreen under the EF1á constitutive promoter. Three days after infection, cells were analyzed by flow cytometry to assess the efficiency of infection. Established iPSC clones were grown in 2i/LIF medium (DMEM/F-12+GlutaMAX, Neurobasal Medium, N2 Supplement-Invitrogen-, B27 supplement-Invitrogen-, 1,5 mM Hepes, 2 mM Glutammine, 0,1 mM β-mercaptoethanol,1/500 home-made LIF, 3 µM CHIR99021, 1 µM PD0325901.

### Infection of TTF and derivation of *Cdkn2a^−/−^*; *Ezh2^fl^* iPSC

Control *Cdkn2a^−/−^Ezh2^fl/+^* and experimental *Cdkn2a^−/−^Ezh2^fl/fl^* TTFs at passage-1 were treated with 50 ug/ml of TAT-Cre recombinase and passaged 3 days later. At 80% confluency, cells underwent a second round of TAT-Cre transduction and were further expanded to allow the dilution of H3K27me3, before infection with STEMCCA and *rtTA* lentiviruses, as described above.

### RNA interference

Puromycin-resistant lentiviral constructs expressing *Eed* shRNAs were purchased from Open Biosystem (TRCN0000095719, TRCN0000095721, TRCN0000095722). Lentiviruses were produced as described above. *Cdkn2a^−/−^* TTFs were infected with viruses expressing either shRNA # TRCN0000095719, TRCN0000095721, TRCN0000095722 or an empty pLKO.1 vector and selected with 2 µg/ml of puromycin. After 6 days of selection, resistant TTFs were reprogrammed by infection with Tet-STEMCCA as described above. *Ezh2^ΔSET/ΔSET^* iPSC clones were infected with viruses expressing either shRNA # TRCN0000095719, TRCN0000095721 or as control, an empty pLKO.1 vector and expanded for two weeks in 0.7 µg/ml of puromycin. RNA, proteins and chromatin were collected on the same day.

### Alkaline phosphatase staining

Alkaline phosphatase staining was performed using the Leukocyte Alkaline Phosphatase kit (Sigma Aldrich) following manufacture's instructions.

### Flow cytometry

One million cells were stained with phycoerythrin-conjugated anti-mouse/human SSEA1 (eBioscience, 12-8813) in FACS buffer (1% bovine serum albumin (BSA), 0,05% NaN_3_ in PBS), washed and acquired on a FACS Calibur instrument (BD Biosciences). Data were analyzed using FlowJo software (Tree Star inc.).

### Teratoma assay

iPSC were cultured in 2i/LIF medium for 3 passages. 2×10^6^ cells were injected subcutaneously into NOD-SCID *Il2rãc^−/−^* mice. 2–3 weeks after injection, mice were sacrificed and tumors were isolated and fixed in 4% formaldehyde for immunohistochemistry.

### RNA extraction and cDNA synthesis

RNA was extracted using TRIzol Reagent (Invitrogen) and purified with RNeasy mini kit (QIAGEN) following manufacturer's instructions. cDNA was prepared using SuperScript VILO cDNA Synthesis Kit (Invitrogen) following manufacturer's instructions.

### Quantitative gene expression analysis

Quantitative real-time PCR analysis for the expression of fibroblast specific genes on MEF, 2 *Ezh2^+/+^* iPSC clones, *2 Ezh2^ΔSET/ΔSET^* iPSC clones, and 1 E14tg2á ESC line was performed using pre-designed Taqman gene expression assays following manufacturer's instructions. (Applied Biosystems: *Cd14*Mm00438094_g1, *Cebpa* Mm00514283_s1, *Dok1* Mm00438532_m1, *Emp2* Mm00801709_m1, *Enpp1* Mm00501097_m1, *Fads3* Mm00517643_m1, *Bmp1*Mm00802220_m1, *Ndrg1* Mm00440447_m1, *Snai2* Mm00441531_m1, *Sox9* Mm00448840_m1, *Tram1l1*, Mm00525200_s1, *Vamp5* Mm00444144_m1, *Bmp1* Mm00802220_m1, *Cap2* Mm00482645_m1, *Vcan* Mm01283063_m1, *Ptprm* Mm00436095_m1, *Lox* Mm00495386_m1, *Pftk1* Mm00448111_m1). Quantitative real-time PCR analysis for the expression of the STEMCCA vector was performed using a custom-designed Taqman gene expression assay, previously described [32].

### cDNA arrays

iPSC were cultured in 2i/LIF medium for 3 passages before RNA extraction. Expression was assessed using the TaqMan Stem Cell Pluripotency Array (Applied Biosystem 4385363) following the manufacturer's instructions. dCT values were calculated subtracting each single CT value to the geometrical mean of the housekeeping genes (*Actb, Raf1, Ctnnb1, Gapdh* and *Eef1a1*). Hierarchical clustering and heat map were obtained using Gene Cluster 3.0 software (Michael Eisen, Copyright 1998–99 Stanford University; Michiel de Hoon, 2002 University of Tokyo, Human Genome Center).

### Microarray analysis

Microarray analysis was performed on 4 *Ezh2^+/+^* iPSC clones, *4 Ezh2^ΔSET/ΔSET^* iPSC clones, 3 independent preparation of MEFs and one sample of E14tg2a ESC using Affymetrix Mouse Gene 1.0 ST arrays. Labeling, hybridization, and washing were performed according to Affimetrix guidelines. Data analysis was performed with R software version 2.15.0 (http://www.r-project.org) starting from raw data (CEL files). Data were normalized using robust multichip analysis (RMA) algorithm [47] as implemented in [48] and made available through Bioconductor (http://www.bioconductor.org/). To perform differential expression analysis, we started from the log_2_ normalized value relative to each gene. Then, we used a t-test to identify significant changes in gene expression between wild type and mutant samples, and a *P* value was calculated for each gene. A false discovery rate (FDR) procedure [49] was applied to take into account multiple testing correction. To identify differentially expressed genes (DEGs) we considered a threshold of 0.05 on the corrected *P* value, along with a cut-off of 1.5-fold changes.

### Gene Ontology analysis

We used Cytoscape software [50] and the BiNGO plugin [51] to analyze Gene Ontology terms significantly enriched in the various gene sets. The enrichment for each term was tested using hypergeometric test and *P* values were corrected using FDR procedure. All terms with a FDR <0.01 were considered enriched.

### Protein extraction and Western blot analysis

iPSC were cultured in 2i/LIF medium for 3 passages before protein extraction. Cell pellets were lysed with urea buffer (8 M Urea, 0,1 M NaH_2_PO_4_, 0,01 M Tris base diluted in water, pH 8.0) at room temperature for 30 minutes on a rotating wheel. Lysates were sonicated with a Bioruptor Sonication System (UCD200) (3 cycles of 30 seconds with one minute breaks, high power). Lysates were centrifuged at 13000 rpm for 15 minutes and supernatants were transferred to a new tube. Protein quantification was performed using Bio-Rad protein assay and following manufacturer's instructions. For the detection of histone modifications 40 µg of total protein extracts were loaded into a 12% acrylamide gel. For the measurement of EZH2 levels, 80 µg of total protein lysates were loaded onto an 8% acrylamide gel. Western blot was performed using standard procedures. Intensities of Western blot bands were determined using ImageJ software (rsbweb.nih.gov/ij/). Antibodies used for Western blot are listed in Table S3.

### Mass spectrometry

A detailed description of core histone extraction and mass spectrometry analysis can be found in Text S1.

### Histology and immunohistochemistry

Teratoma tissues were washed in PBS buffer for 30 minutes, fixed in 4% buffered formalin for 4 hours and paraffin embedded. Samples were processed in consecutive 3 µm thick sections and stained with haematoxylin and eosin. Immunostaining for desmin, protein S-100 and cytokeratin were performed using an automated immunostainer (Autostainer, DakoCytomation, Glostrup, Denmark) and a commercially available detection kit (DakoEnVision Plus-HRP), according to the manufacturer's instructions. Detailed information about antibodies and immunostaining procedure are listed in Table S4. After immunostaining, sections were counterstained with 1% modified Harris hematoxylin, dehydrated and mounted. Standard reference positive and negative controls were run simultaneously. Haematoxylin and eosin and immunohistochemistry images were taken with an Olympus Upright BX 51 optical microscope equipped with a Nikon digital color camera. Digital images were processed with Adobe Photoshop CS3.

### Chromatin immunoprecipitation

iPSC cultured in 2i/LIF medium were trypsinized and resuspended in 1% formaldehyde/PBS solution. Cross-linking was allowed to proceed for 10 min at room temperature and stopped by addition of glycine at a final concentration of 0.125 M, followed by an additional incubation for 5 min. Fixed cells were washed twice with PBS and resuspended in SDS buffer (SDS 0.5%, Tris-Cl pH 8.1 50 mM, NaCl 100 mM, EDTA pH 8 5 mM, NaN_3_ 0.02%, diluted in ddH_2_O) and stored at −80°C. SDS cell suspensions were thawed at room temperature using a water bath for 20 minutes, centrifuged at 2000 rpm and resuspended in ice cold IP buffer (SDS buffer/Triton dilution buffer = 2∶1; Triton dilution buffer: Triton X-100 5%, Tris-Cl pH 8.6 100 mM, NaCl 100 mM, EDTA pH 8 5 mM, NaN_3_ 0.02%, diluted in ddH_2_O). Fixed cells were sonicated yielding genomic DNA fragments with a bulk size of 400 to 1000 bp. Sonicated material was centrifuged at 13000 rpm for 30 minutes at 4°C and supernatants were transferred into a new tube. Chromatin was quantified using Bio-Rad protein assay following manufacturer's instructions. For immunoprecipitation using antibodies against H3 and specific histone modifications, 100 µg of chromatin were used. For immunoprecipitation of SUZ12, 500 µg of chromatin were used. For each immunoprecipitation assay, chromatin was diluted in 1 ml IP buffer and 10 µl were taken and stored at −80°C as 1% of input. Primary antibodies were incubated overnight at 4°C on a rotating platform. To each sample, 50 µl of 50% slurry of protein A-Sepharose (Amersham) beads were added for 2–3 h. Beads were washed three times in 150 mM wash buffer and one time in 500 mM wash buffer (Triton-X 1%, NaCl 150 mM or 500 mM, Tris-Cl pH 8.0 20 mM, SDS 0.1%, EDTA pH8 2 mM diluted in water). Beads (and input samples) were resuspended in 120 µl of 0.1% SDS, 0.1 M NaHCO3 buffer and de-cross-linked at 65°C overnight. DNA was purified using PCR purification kit (QIAGEN) following the manufacturer's instruction and eluted in 100 µl of water. 1 µl of eluted material was used for each real-time quantitative PCR (qPCR) reaction. Quantitative real-time PCR analysis was performed in triplicate using Fast SYBR Green master mix (Applied Biosystems) in a 7500 Fast Real-Time PCR instrument (Applied Biosystems). Antibodies used for ChIP are listed in Table S3. Primers used for ChIP-qPCR are listed in Table S5.

### ChIP–seq

ChIP-seq analysis was performed on two *Ezh2* ^+/+^ iPSC clones and two *Ezh2^ΔSET/ΔSET^* iPSC clones. 10 ng of sheared DNA obtained before (input) and after chromatin IP using anti-H3K27me3 and -H3K27me2 specific antibodies, were prepared with the Illumina ChIPSeq sample prep kit and multiplexing oligonucleotide kit. DNA libraries were quantified using a high sensitivity Chip on Bioanalyzer (Agilent) and diluted to a concentration of 16 pM. Diluted libraries were used for cluster generation and sequencing on a HiSeq 2000 instrument (Illumina) following manufacturer's protocol.

### Bioinformatics analysis of ChIP–seq data

#### Mapping of reads

After filtering for artifacts with FASTX-Toolkit v.0.0.13, reads were aligned to the mm9 genome using Bowtie v.0.12.7 [52], allowing up to two mismatches per read and discarding multiply-aligning reads. Each ChIP-seq experiment had a total of 18 to 28 millions of uniquely mapped reads. Aligned reads of a MEF H3K27me3 ChIP-seq dataset were downloaded from the Gene Expression Omnibus (accession id GSM656316) and processed similarly to the other datasets (see below).

#### Identification of enriched domains

We used two different strategies to identify enriched domains using, respectively, MACS v.1.4.0 [53] and RSEG v.0.4.8 [54]. For MACS analysis, we disabled the shifting model and the dynamic lambda (which are inappropriate for histone modifications) and used a stringent *p-value* threshold of 10e-10. A more generous identification of enriched regions was done using RSEG, which was specifically designed to identify extended regions marked by histone marks (RSEG was independently benchmarked in [55]). We used Hideaki's empirical method to determine bin size, 20 iterations for the training, and provided deadzones (regions where no read can map uniquely) as described in the documentation. Domains called as unconfident were discarded. In both approaches, we compared the signal over the input.

On the basis of a calibration performed in ESC and of biological and technical considerations (see Text S1 for further details), we applied the MACS method to identify H3K27me3 enriched genomic regions, while RSEG was used to identify domains of enriched H3K27me2.

#### Annotation

For both H3K27me3 and H3K27me2, we assigned enriched regions to genes by determining for each RefSeq transcript, whether an enriched domain overlapped with a +/−5 kb region around the transcription start site. We also tested a +/−2.5 kb interval, which resulted in a marginal decrease in the number of gene annotations. Transcripts were then collapsed to gene symbols using BioMart [56], and only the genes marked in both biological replicates (Figure S4D) were retained for further analysis.

Heat map and clustering of H3K27me3 and H3K27me2 distributions around the TSS were produced with Seqminer [57] using K-means linear normalization.

Venn diagrams showing overlap between different datasets were prepared using BioVenn (by Tim Hulsen http://www.cmbi.ru.nl/cdd/biovenn/).

### Ethics statement

This project involved the minimum number of mice required to fulfil the research objectives. Experiments involving animals were performed in accordance with the Italian Laws (D.L.vo 116/92 and following additions), which enforces EU 86/609 Directive (Council Directive 86/609/EEC of 24 November 1986 on the approximation of laws, regulations and administrative provisions of the Member States regarding the protection of animals used for experimental and other scientific purposes). The authority responsible for ensuring compliance with the provisions of EU 86/609 Directive is the Italian Ministry of Health. Our mouse facility is authorized by the Ministry of Health (DM N°86/2005 - 17/06/2005) and a veterinarian is responsible for the well-being of the experimental animals.

An Institutional Animal Care and Use Committee (IACUC) of the IFOM Foundation-FIRC Institute of Molecular Oncology Foundation, supervises the ethical conduct of research involving non-human vertebrates.

## Supporting Information

Figure S1 Molecular and biochemical characterization of *Ezh2* mutant iPSC clones. A. Flow cytometric analysis of *Ezh2* control (+/*Δ*SET, upper row) and mutant (*Δ*SET/*Δ*SET, lower row) MEF infected with STEMCCA, *rtTA* and GFP-expressing lentiviruses in two replicate experiments (left and right panel). MEF are gated according to cell size (FSC) and GFP expression. Numbers within dot plots indicate percentage of gated cells. B. Table indicating the status of the *Ezh2* floxed allele in individual iPSC clones isolated upon reprogramming of TAT-Cre transduced *Ezh2^fl/+^* and *Ezh2^fl/fl^* MEF. Numbers of iPSC clones of the indicated *Ezh2* genotypes and total number of isolated clones are shown. C. Western blot analysis of H3K9me3 levels in embryonic stem cells (ESC), control (+/+ and *Δ*SET/+) and mutant (*Δ*SET/*Δ*SET) iPSC (two clones/genotype). H3 levels were used as control for protein loading. D. Transcription from the STEMCCA vector as revealed by qRT-PCR in representative control (n = 4) and mutant (n = 3) iPSC clones. Measurements are relative to STEMCCA transcript levels detected in infected MEF treated for 10 days with doxycycline. Uninfected MEF were used as negative control. Standard deviations refer to replicates of the q-PCR reaction. E. Annotated MS/MS spectra of H3 peptide 27–40 species, with one or more co-existing post-translational modifications detected in *Ezh2* control (+/+; left spectra) and mutant (*Δ*SET/*Δ*SET) iPSC. Spectra are displayed according to whether the peptide contained a total of 3 (upper row), 4 (middle row) or 5 (lower row) methyl groups. The m/z ratio of b- and y- product ions identified are annotated in the spectrum and also reported along the amino acid sequence in blue and red, respectively.
(TIF)

Figure S2 Identification of MEF specific genes by cDNA microarray analysis. Heat map representation of the average expression profile of MEF coming from 3 different embryos, 4 *Ezh2* control (+/+) iPSC clones and 4 *Ezh2* mutant (*Δ*SET/*Δ*SET) iPSC clones. Shown are the expression levels of 3644 genes differentially expressed between iPSCs and MEFs (p-value = 0.05; f.c = 1.5). Expression ranges from lower (green) to higher levels (red).
(TIF)

Figure S3 Establishment of iPSC clones upon genome-wide erasure of H3K27me3 at the onset of reprogramming. A. H3K27me3 and p16^Ink4a^ protein levels measured by Western blot analysis in representative populations of TAT-cre transduced *Ezh2* control (+/*Δ*SET) and mutant (*Δ*SET/*Δ*SET) MEF after two passages and 11 days of culture. As comparison, representative control (+/+) and mutant (*Δ*SET/*Δ*SET) iPSC clones were analyzed. Vinculin protein levels were used as loading control. B. Flow cytometric assessment of the infection efficiency of *Ezh2* control (*Ezh2^Δ^* ^SET/+^; right columns) and mutant (*Ezh2^Δ^* ^SET/*Δ*SET^, left columns) *Cdkn2a^−/−^* TTFs (3 independent batches/genotype). Cells were infected with STEMCCA, *rtTA* and GFP expressing lentiviruses. Numbers within plots indicate percentage of gated cells.
(TIF)

Figure S4 Epigenetic characterization of *Ezh2^ΔSET/ΔSET^* iPSCs. A. Analysis of transcript levels (qRT-PCR) and status of PRC2 (SUZ12), H3K27me2 and H3K27me1 (ChIP q-PCR) enrichment at promoters of 17 genes overexpressed in MEF vs. iPSC. For all analyses, two *Ezh2* control (+/+; grey) iPSC clones were compared to two mutant (*Δ*SET/*Δ*SET; purple) counterparts. Levels of expression are shown as ddCt (log_2_ scale) relative MEF. Status of a particular histone modification (±SEM) is represented as enrichment relative to input, after normalization for H3 density within the same amplicon. SUZ12 enrichment at promoters of the indicated genes is assessed comparing it to that of unrelated IgG. Error bars referred to qPCR triplicates. B. Distribution of the maximum height of H3K27me3 signal in *Ezh2^+/+^* iPSC according to H3K27me status in 2 *Ezh2^ΔSET/ΔSET^* iPSC clones: genes that lose H3K27me3 (red lines), genes that retain H3K27me3 (green lines). P-value<2.2e-16 (two-sided t-test). C. Heat map representation of H3K27me3 and H3K27me2 distributions in a ±5 Kb window around the TSS of genes marked by at least one of the two methylation states. Data from 2 representative clones per genotype are shown Regions were inverted depending on the direction of transcription. Genes were clustered according to the combination of the two marks. D. Venn diagrams showing overlap of H3K27me3 targets (upper row) or H3K27me2 targets (lower row) between 2 iPSC clones of the same genotype (*Ezh2* ^+/+^ left; *Ezh2^ΔSET/ΔSET^* right).
(TIF)

Figure S5 Targets of H3K27me3 in *Ezh2* mutant iPSC are enriched for transcriptional regulators and developmental determinants. A. Tree diagram representing the main steps that lead to the identification of 175 genes that acquired H3K27me3 *de novo* in the MEF to iPSC transition. See main text for further explanation. B Gene ontology analysis of *de novo* H3K27 trimethylated genes in the MEF to iPSC transition. Bars represent P values in –Log_2_ scale of the corresponding biological process. Dashed line indicates significance threshold. C. Network showing protein-DNA interactions (blue lines) between genes that change expression upon reprogramming (blue circles) and the four identified master regulators of fibroblast expression program (yellow circles). D. qRT-PCR analysis of *Ets1* and *Egr1* transcript levels at day 0, day 3, day 6, day 10 and day 14 of MEF reprogramming. mRNA levels are normalized to *Tbp*. Error bars represents qPCR triplicates.
(TIF)

Figure S6 TF–induced reprogramming in the absence of PRC2. A. Experimental time line showing the main experimental steps: Infection of *Ezh2* proficient *Cdkn2a^−/−^* TTFs with control virus (empty) or lentiviruses expressing independent short hairpin (sh) RNAs targeting *Eed*; puromycin selection; Infection with Tet-STEMCCA and *rtTA* lentiviruses; replating of infected cells at cloning dilution and addition of ESC medium supplemented with doxycycline; Doxycycline withdrawal and scoring of AP-positive colonies.
(TIF)

Table S1 qRT-PCR analysis of transcript levels in two representative *Ezh2^+/+^* (# 40A7 and # 40A8) and *Ezh2^ΔSET/ΔSET^*(# 48B4 and # 48B7) iPSC clones, of a selected list of genes controlling stem cell pluripotency, self renewal and differentiation. Transcript levels were also measured in ESC and MEF. Data are represented as dCts relative to the geometric mean of 5 housekeeping genes.
(XLSX)

Table S2 qPCR results of H3, H3K4me3, H3K27me3, H3K27me2, and H3K27me1 ChIP on promoters of 17 MEF-specific genes in two representative *Ezh2* control (+/+, grey bars) and mutant (*Δ*SET/*Δ*SET, purple bars) iPSC clones. Data are represented as fold change of input. The same analysis was performed using unrelated IgG for the ChIP assays. Averages and standard deviation refers to qPCR triplicates.
(XLSX)

Table S3 List of antibodies used for Western blot, ChIP, and flow cytometry.
(DOCX)

Table S4 List of antibodies used for immunohistochemistry.
(DOCX)

Table S5 List of primers used for ChIP–qPCR and genotyping.
(DOCX)

Text S1 Supporting information on ChIP–seq and mass spectrometry analyses.
(DOCX)
