# Supplementary figures and images for: Cell Reprogramming Requires Silencing of a Core Subset of Polycomb Targets
Source: PLoS Genet. 2013 Feb 28;9(2):e1003292. doi: 10.1371/journal.pgen.1003292 (PMC3585017; doi:10.1371/journal.pgen.1003292)

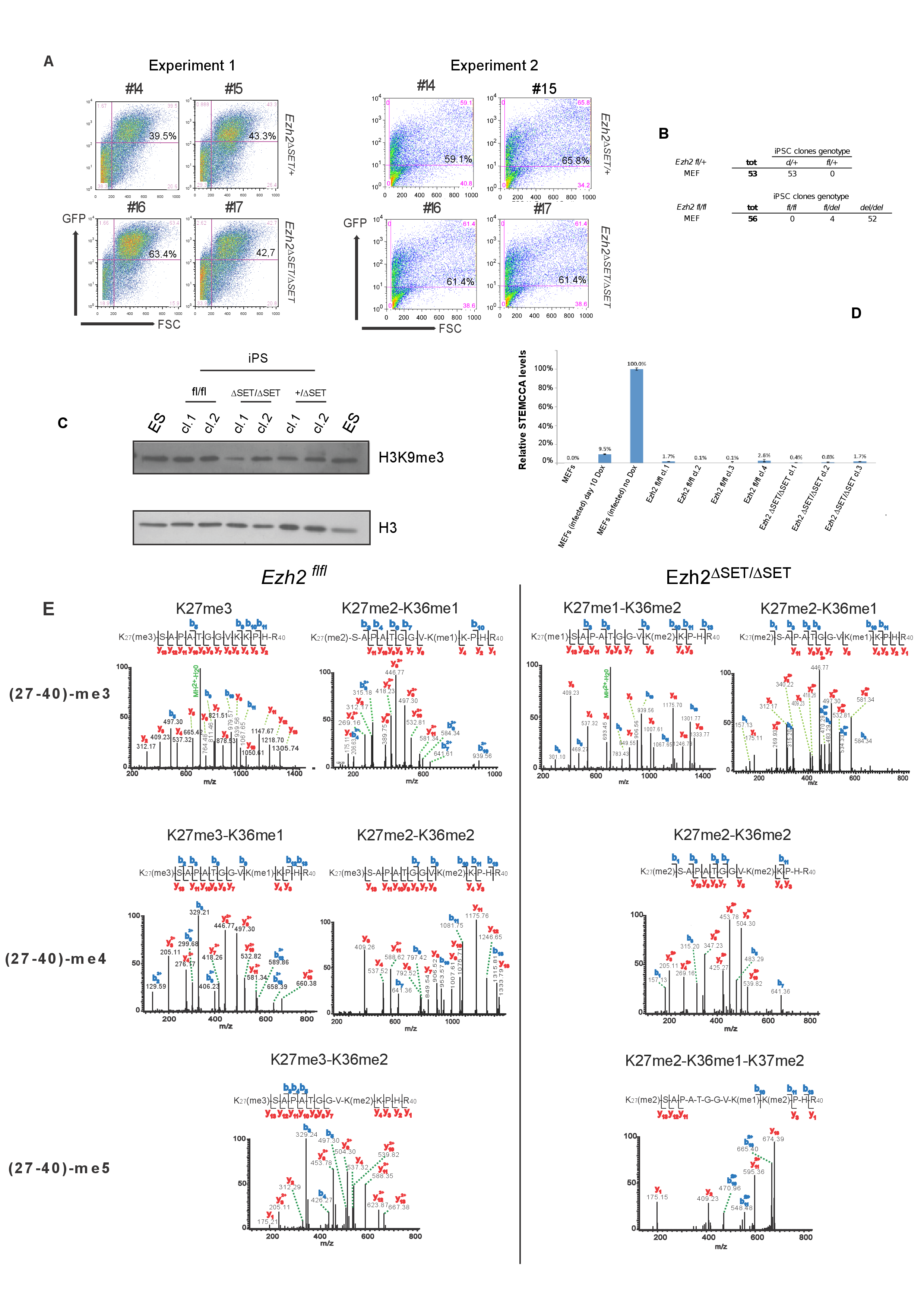

Supplement: Figure S1 — Molecular and biochemical characterization of Ezh2 mutant iPSC clones. A. Flow cytometric analysis of Ezh2 control (+/ΔSET, upper row) and mutant (ΔSET/ΔSET, lower row) MEF infected with STEMCCA, rtTA and GFP-expressing lentiviruses in two replicate experiments (left and right panel). MEF are gated according to cell size (FSC) and GFP expression. Numbers within dot plots indicate percentage of gated cells. B. Table indicating the status of the Ezh2 floxed allele in individual iPSC clones isolated upon reprogramming of TAT-Cre transduced Ezh2fl/+ and Ezh2fl/fl MEF. Numbers of iPSC clones of the indicated Ezh2 genotypes and total number of isolated clones are shown. C. Western blot analysis of H3K9me3 levels in embryonic stem cells (ESC), control (+/+ and ΔSET/+) and mutant (ΔSET/ΔSET) iPSC (two clones/genotype). H3 levels were used as control for protein loading. D. Transcription from the STEMCCA vector as revealed by qRT-PCR in representative control (n = 4) and mutant (n = 3) iPSC clones. Measurements are relative to STEMCCA transcript levels detected in infected MEF treated for 10 days with doxycycline. Uninfected MEF were used as negative control. Standard deviations refer to replicates of the q-PCR reaction. E. Annotated MS/MS spectra of H3 peptide 27–40 species, with one or more co-existing post-translational modifications detected in Ezh2 control (+/+; left spectra) and mutant (ΔSET/ΔSET) iPSC. Spectra are displayed according to whether the peptide contained a total of 3 (upper row), 4 (middle row) or 5 (lower row) methyl groups. The m/z ratio of b- and y- product ions identified are annotated in the spectrum and also reported along the amino acid sequence in blue and red, respectively. (TIF) [file pgen.1003292.s001.tif]

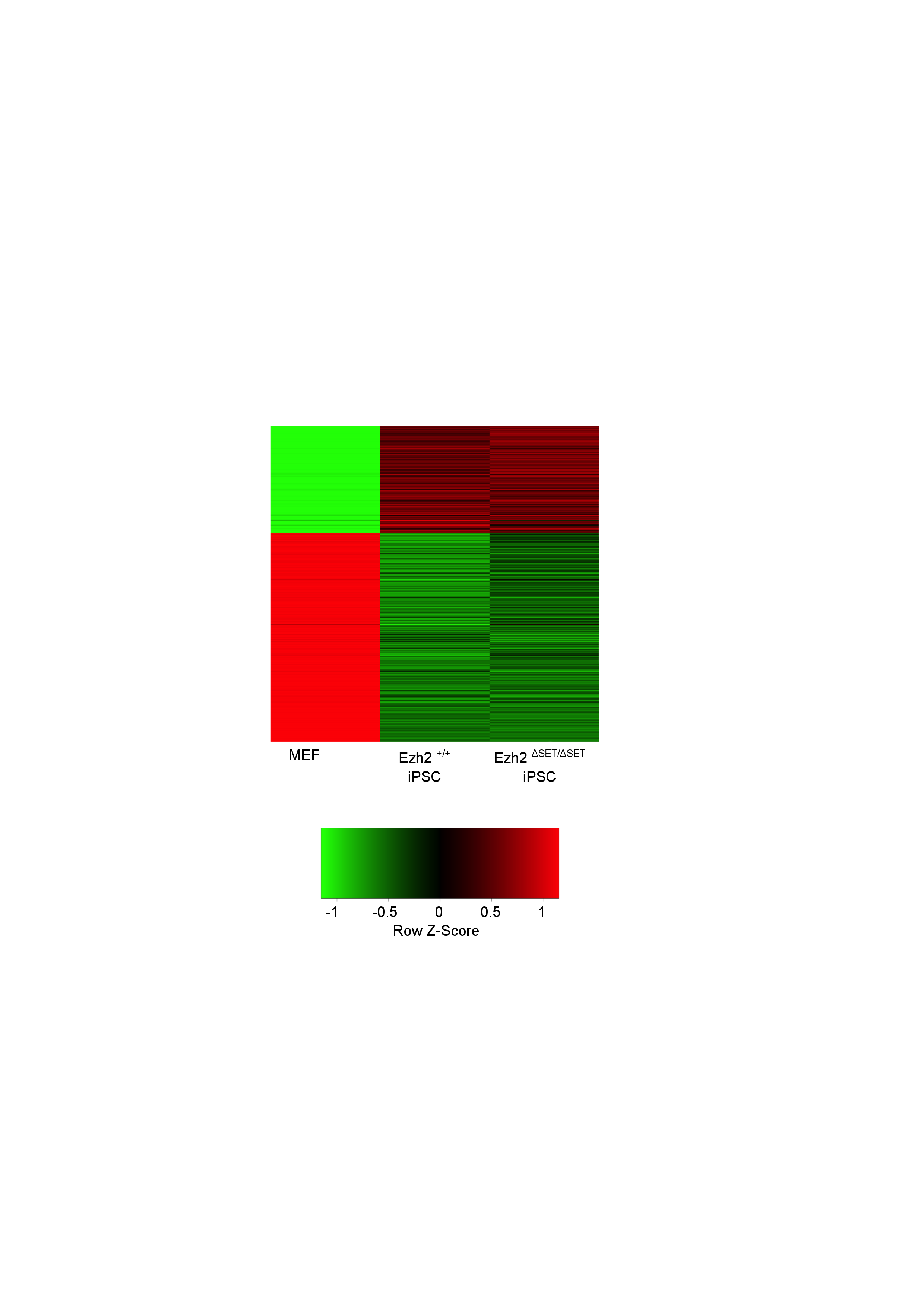

Supplement: Figure S2 — Identification of MEF specific genes by cDNA microarray analysis. Heat map representation of the average expression profile of MEF coming from 3 different embryos, 4 Ezh2 control (+/+) iPSC clones and 4 Ezh2 mutant (ΔSET/ΔSET) iPSC clones. Shown are the expression levels of 3644 genes differentially expressed between iPSCs and MEFs (p-value = 0.05; f.c = 1.5). Expression ranges from lower (green) to higher levels (red). (TIF) [file pgen.1003292.s002.tif]

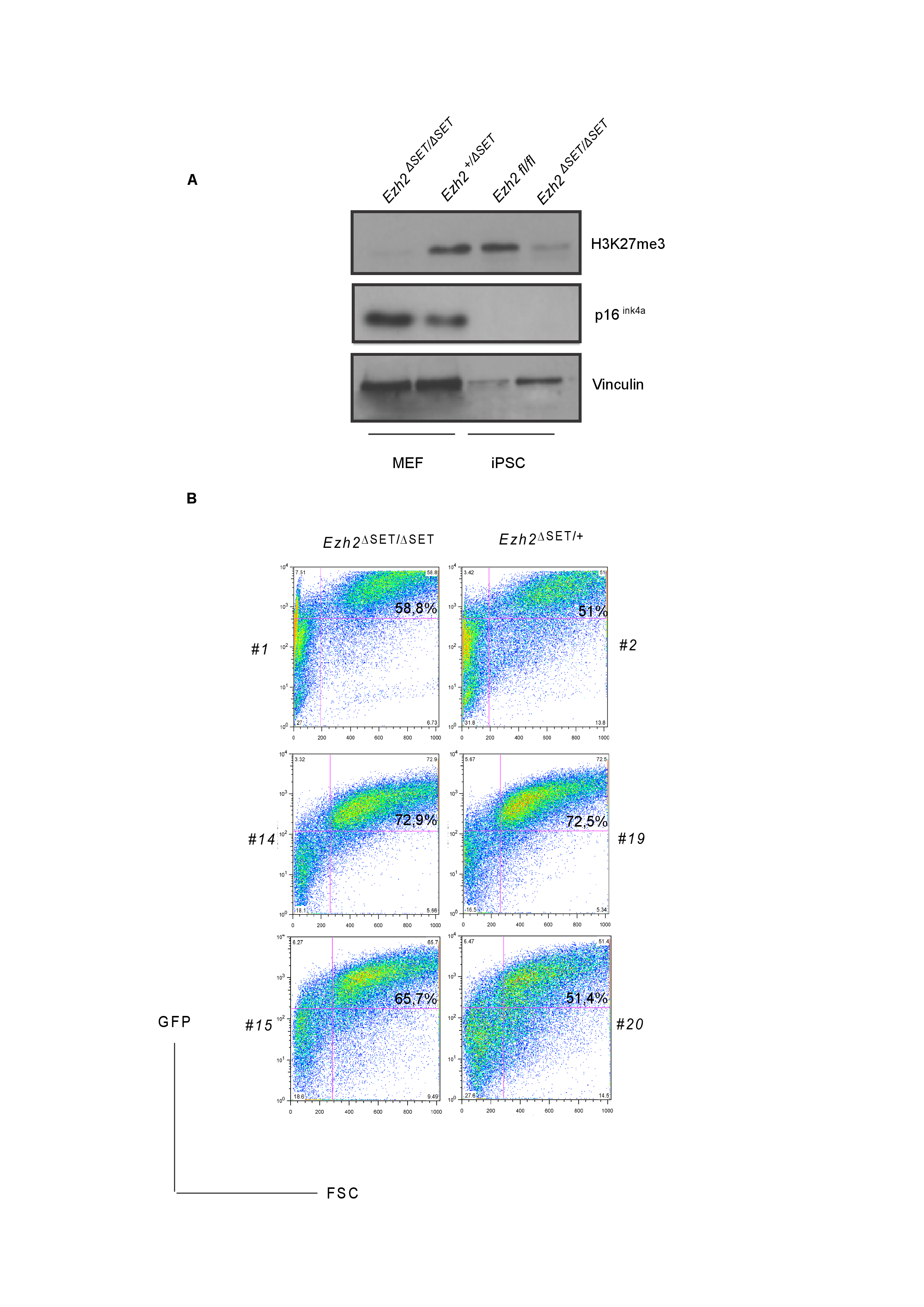

Supplement: Figure S3 — Establishment of iPSC clones upon genome-wide erasure of H3K27me3 at the onset of reprogramming. A. H3K27me3 and p16Ink4a protein levels measured by Western blot analysis in representative populations of TAT-cre transduced Ezh2 control (+/ΔSET) and mutant (ΔSET/ΔSET) MEF after two passages and 11 days of culture. As comparison, representative control (+/+) and mutant (ΔSET/ΔSET) iPSC clones were analyzed. Vinculin protein levels were used as loading control. B. Flow cytometric assessment of the infection efficiency of Ezh2 control (Ezh2Δ SET/+; right columns) and mutant (Ezh2Δ SET/ΔSET, left columns) Cdkn2a−/− TTFs (3 independent batches/genotype). Cells were infected with STEMCCA, rtTA and GFP expressing lentiviruses. Numbers within plots indicate percentage of gated cells. (TIF) [file pgen.1003292.s003.tif]

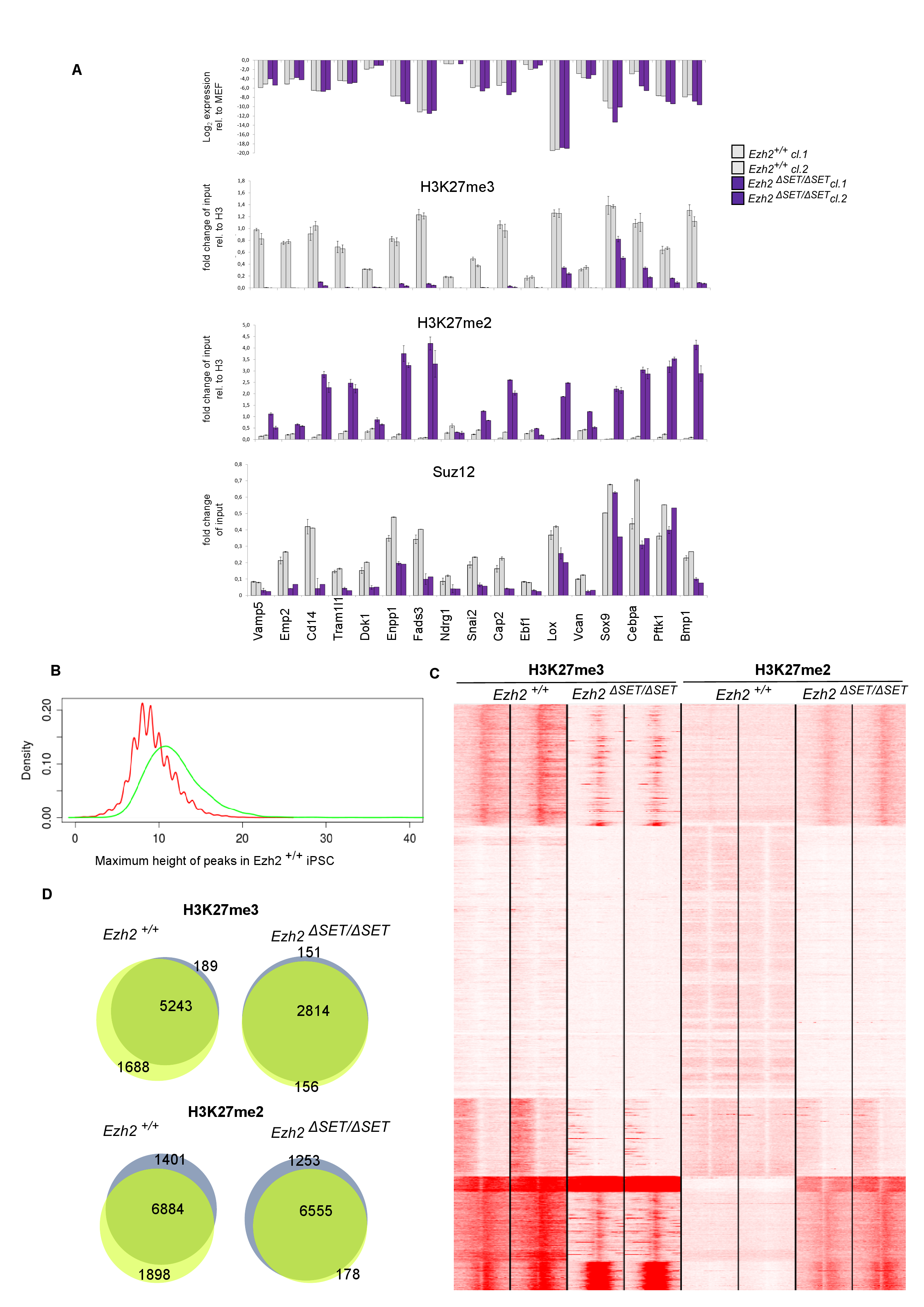

Supplement: Figure S4 — Epigenetic characterization of Ezh2ΔSET/ΔSET iPSCs. A. Analysis of transcript levels (qRT-PCR) and status of PRC2 (SUZ12), H3K27me2 and H3K27me1 (ChIP q-PCR) enrichment at promoters of 17 genes overexpressed in MEF vs. iPSC. For all analyses, two Ezh2 control (+/+; grey) iPSC clones were compared to two mutant (ΔSET/ΔSET; purple) counterparts. Levels of expression are shown as ddCt (log2 scale) relative MEF. Status of a particular histone modification (±SEM) is represented as enrichment relative to input, after normalization for H3 density within the same amplicon. SUZ12 enrichment at promoters of the indicated genes is assessed comparing it to that of unrelated IgG. Error bars referred to qPCR triplicates. B. Distribution of the maximum height of H3K27me3 signal in Ezh2+/+ iPSC according to H3K27me status in 2 Ezh2ΔSET/ΔSET iPSC clones: genes that lose H3K27me3 (red lines), genes that retain H3K27me3 (green lines). P-value<2.2e-16 (two-sided t-test). C. Heat map representation of H3K27me3 and H3K27me2 distributions in a ±5 Kb window around the TSS of genes marked by at least one of the two methylation states. Data from 2 representative clones per genotype are shown Regions were inverted depending on the direction of transcription. Genes were clustered according to the combination of the two marks. D. Venn diagrams showing overlap of H3K27me3 targets (upper row) or H3K27me2 targets (lower row) between 2 iPSC clones of the same genotype (Ezh2 +/+ left; Ezh2ΔSET/ΔSET right). (TIF) [file pgen.1003292.s004.tif]

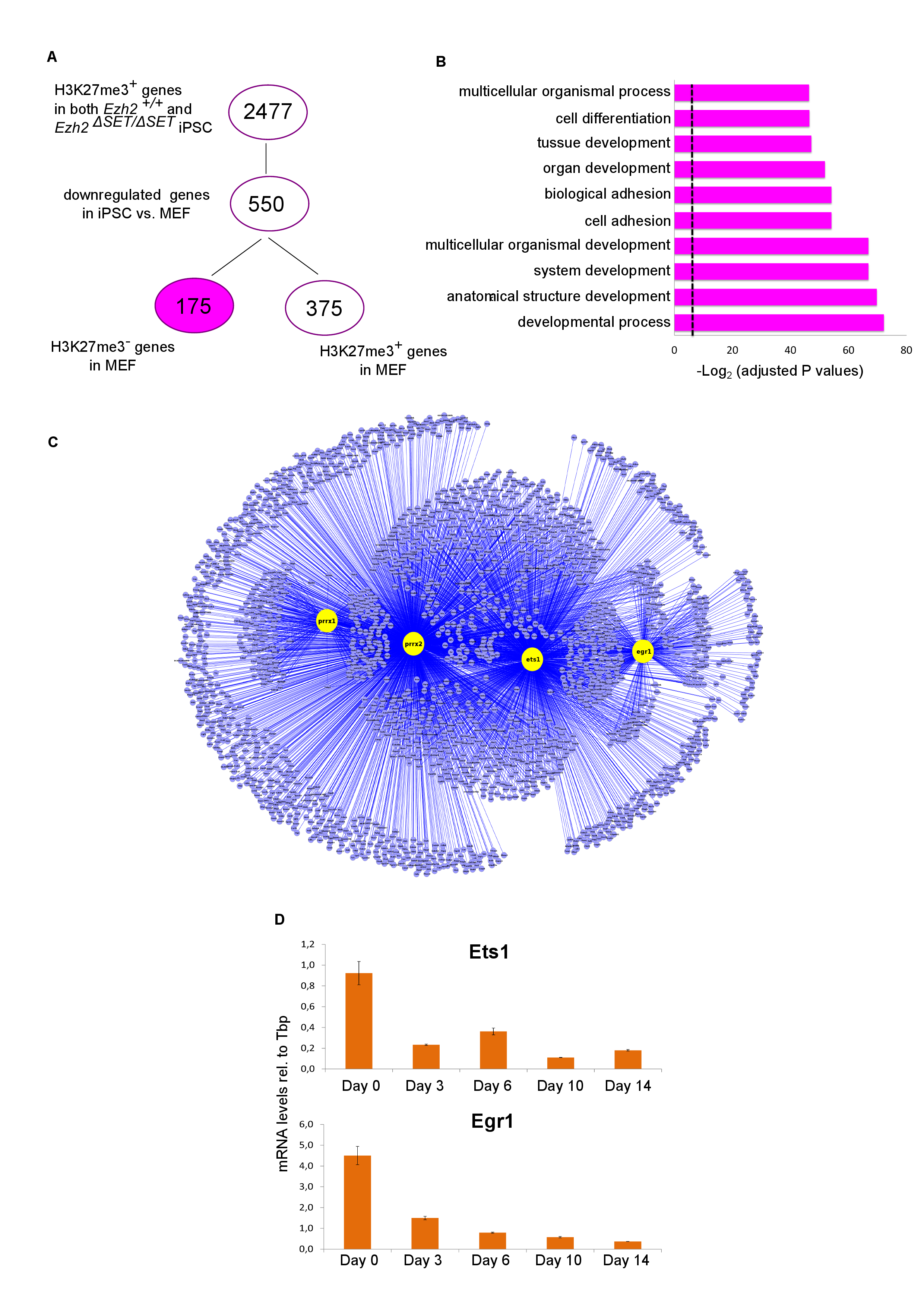

Supplement: Figure S5 — Targets of H3K27me3 in Ezh2 mutant iPSC are enriched for transcriptional regulators and developmental determinants. A. Tree diagram representing the main steps that lead to the identification of 175 genes that acquired H3K27me3 de novo in the MEF to iPSC transition. See main text for further explanation. B Gene ontology analysis of de novo H3K27 trimethylated genes in the MEF to iPSC transition. Bars represent P values in –Log2 scale of the corresponding biological process. Dashed line indicates significance threshold. C. Network showing protein-DNA interactions (blue lines) between genes that change expression upon reprogramming (blue circles) and the four identified master regulators of fibroblast expression program (yellow circles). D. qRT-PCR analysis of Ets1 and Egr1 transcript levels at day 0, day 3, day 6, day 10 and day 14 of MEF reprogramming. mRNA levels are normalized to Tbp. Error bars represents qPCR triplicates. (TIF) [file pgen.1003292.s005.tif]

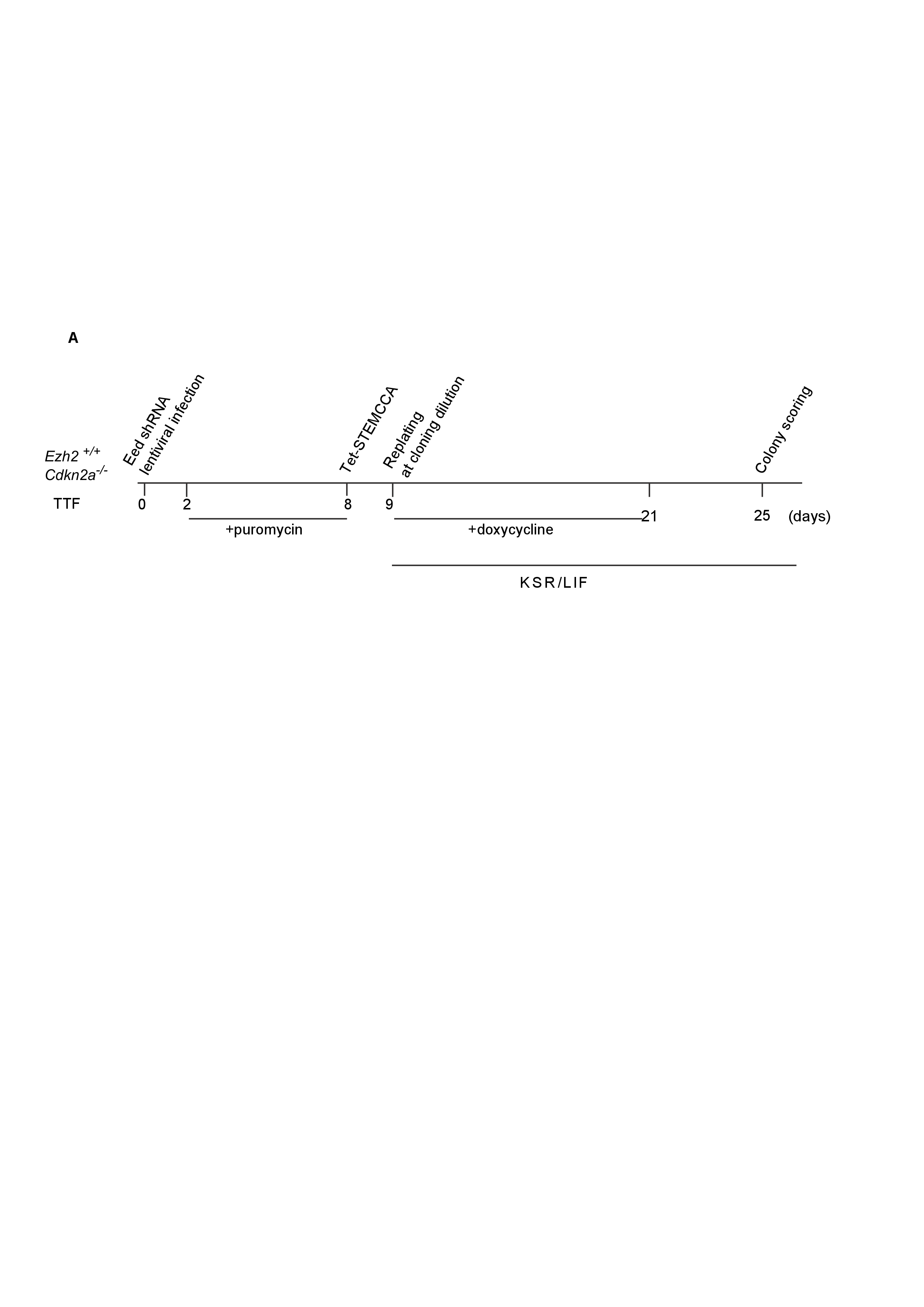

Supplement: Figure S6 — TF–induced reprogramming in the absence of PRC2. A. Experimental time line showing the main experimental steps: Infection of Ezh2 proficient Cdkn2a−/− TTFs with control virus (empty) or lentiviruses expressing independent short hairpin (sh) RNAs targeting Eed; puromycin selection; Infection with Tet-STEMCCA and rtTA lentiviruses; replating of infected cells at cloning dilution and addition of ESC medium supplemented with doxycycline; Doxycycline withdrawal and scoring of AP-positive colonies. (TIF) [file pgen.1003292.s006.tif]
