# Supplementary material for: Cell Reprogramming Requires Silencing of a Core Subset of Polycomb Targets
Source: PLoS Genet. 2013 Feb 28;9(2):e1003292. doi: 10.1371/journal.pgen.1003292 (PMC3585017; doi:10.1371/journal.pgen.1003292)
Supplement: Table S3 — List of antibodies used for Western blot, ChIP, and flow cytometry. (DOCX) [file pgen.1003292.s009.docx]

| **Antibodies for ChIP, Western blot and flow cytometry** | | | | |
| --- | --- | --- | --- | --- |
| Name | Type | Cat # | Company | Application |
| Anti-trimethyl-Histone H3  (Lys 4) | Rabbit, monoclonal,  clone Mc315 | 04-745 | Millipore | ChIP |
| Anti-monomethyl-Histone H3 (Lys27) | Rabbit,  polyclonal | 07-488 | Millipore | ChIP, Western blot |
| Anti-dimethil-Histone H3 (Lys27) | Rabbit,  monoclonal,  clone D18C8 | 9728 | Cell Signaling | ChIP, Western blot |
| Anti-trimethyl-Histone H3 (Lys27) | Rabbit,  Monoclonal,  Clone C36B11 | 9733 | Cell Signaling | ChIP, Western blot |
| Anti-Histone H3 | Rabbit,  polyclonal | 07-690 | Upstate | Western blot |
| Anti-Histone H3 | Rabbit,  Polyconal | 1791 | Abcam | ChIP |
| Anti-trimethyl Histone H3 (Lys9) | Rabbit,  Polyconal | 07-442 | Millipore | Western blot |
| Anti-Suz12 | Rabbit,  Monoclonal,  Clone D39F6 | 3737 | Cell Signaling | ChIP |
| Anti-Ezh2 | Mouse,  Monoclonal,  Clone AE25-13 |  | Home-made | Western blot |
| Anti-Eed | Mouse,  Monoclonal,  Clone AA19.30 |  | Home-made | Western blot |
| Pe-conjugated anti-SSEA1 | Mouse,  Monoclonal,  MC480 | 12-8813 | eBioscience | Flow cytometry |
