# Supplementary material for: Cell Reprogramming Requires Silencing of a Core Subset of Polycomb Targets
Source: PLoS Genet. 2013 Feb 28;9(2):e1003292. doi: 10.1371/journal.pgen.1003292 (PMC3585017; doi:10.1371/journal.pgen.1003292)
Supplement: Table S4 — List of antibodies used for immunohistochemistry. (DOCX) [file pgen.1003292.s010.docx]

| **Antibodies for Immunohistochemistry** | | | | | |
| --- | --- | --- | --- | --- | --- |
| Name | Type | Antigen unmasking | Working dilution | Incubation  conditions | Company |
| Anti-Desmin | Mouse,  Monoclonal,  Clone D33 | EDTA 0.25mM pH 8  30 min.  95°C | 1:20 | 30 min.  Room temperature | Dako, M0760 |
| Anti-S100 | Rabbit,  Polyclonal | none | 1:1600 | 30 min.  Room temperature | Dako, Z0311 |
| Anti-Cytokeratin | Muse,  monoclonal,  clone MNF 116 | pepsin  5 min.  37°C | 1:400 | 30 min.  Room temperature | Dako, M0821 |
