# Supplementary material for: Cell Reprogramming Requires Silencing of a Core Subset of Polycomb Targets
Source: PLoS Genet. 2013 Feb 28;9(2):e1003292. doi: 10.1371/journal.pgen.1003292 (PMC3585017; doi:10.1371/journal.pgen.1003292)
Supplement: Table S5 — List of primers used for ChIP–qPCR and genotyping. (DOCX) [file pgen.1003292.s011.docx]

| **ChIP-qPCR primers** | | | |
| --- | --- | --- | --- |
| Gene | | Forward | Reverse |
| *Bmp1* | | TTCCTGTCCCGCTGCGGTTTCTAC | AAAGTCAGTGGTCTCGGGAGCTCG |
| *Cap2* | | TGAGCTTTCTGAGGGTTCCCGG | TTCGGATTGAGGTGGCACTGGC |
| *Cd14* | | TTGTTGCCCACGACACGTTGCG | TTTCCGGCCTCCAGGAACTGACTC |
| *Cebpa* | | TTGACTAGAGTGCTCCACGCTGGG | TCCAGGCCAGAGCGATAGGATTGC |
| *Dok1* | | GAACGAAAGACTCGAGAGTGCGCG | AAAGTAGCTCAGCCTGGGAAGCCC |
| *Ebf1* | | ACTTTGCCGGGAACTTTGGCGG | GAGCTGGCTTGTTCACAGAGCCTG |
| *Emp2* | | GCCGTCCAGCAGCACTCTGTAAAG | TGAGAAGGGCAGCGATGATCCCAG |
| *Enpp1* | | TGGCTTCCTGTCTGATTTCGGGCC | ATTCCGCAAGGGTTCGTAGGGTGC |
| *Fads3* | | AGATCCGCCAGCATGACCTACCAG | TGTGGGTTAGCTTACCGTGGCGTC |
| *Lox* | | TGCAGTTACACAAGCCGTTCTGGC | TCCGCCTTGCACGTTTCCAATCAC |
| *Ndrg1* | | CACGGAAAGGAGCCAACCACAAGG | TCGCGTTGCAGGCAGATTGCTC |
| *Pftk1* | | CACTCACCGATGCGGCTGAAACTC | ATGTGCGACCTCATTGAACCGCAG |
| *Ptprm* | | GTTTGCCAAGCATCCACGTGCG | AAGGTGGGTGGCACAGAGTAGCAG |
| *Snai2* | | GCCCAACTACAGCGAACTGGACAC | CTCGCGCACAGATTTACAGAGGCG |
| *Sox9* | | AACCGACGTGCAAGCTGGCAAAG | GTCGCTGCTCAGTTCACCGATGTC |
| *Tram1l1* | | GTCTCCTGCGTGGGCATGTTCTTC | TGAGGAACGCGATCGACATCTCGG |
| *Vamp5* | | AGTTAGCCCAGGAGGCGTCATTCC | AGCTCCAGCACATAGCTCCCACAG |
| *Vcan* | | AGGAGCTGCCCAGAGGTGACTTTC | TTTCCTGGCTCCGTGTGTGTGGTC |
| *Pdgfra* | | CCAGCCTTGGCGAGAAAGGAAGTG | TTTCTAAGCGCGGGTTCTGCAAGG |
| **Primer for genotyping** | | | |
| Allele | Forward | | Reverse |
| *Oct4-GFP* | AAGGCAAGGGAGGTAGACAA | | AGGAACTGCTTCCTTCACGA |
|  |  | | TGCCAGACAATGGCTATGAG |
| *Ezh2* | ACACACCCGACTGGCTTTAC | | ATGGGCCTCATAGTGACAGG |
|  |  | | ACGAAACAGCTCCAGATTCAGGG |
| *Cdkn2a* | TAACAGCGGAGCTTCGTACA | | TGCACCGTAGTTGAGCAGAA |
|  |  | | GCCTACCCGCTTCCATTGCT |
