# Supplementary material for: Cell Reprogramming Requires Silencing of a Core Subset of Polycomb Targets
Source: PLoS Genet. 2013 Feb 28;9(2):e1003292. doi: 10.1371/journal.pgen.1003292 (PMC3585017; doi:10.1371/journal.pgen.1003292)
Supplement: Text S1 — Supporting information on ChIP–seq and mass spectrometry analyses. (DOCX) [file pgen.1003292.s012.docx]

**Supporting Text S1**

**Selection of the method of choice for the identification of genome wide targets of H3K27me3 and H3K27me2 in iPSC clones.**

RSEG and MACS methods (described in the Materials and Methods section) identified highly similar genomic regions marked by H3K27me3 in *Ezh2^∆SET/∆SET^* iPSC clones. RSEG identified considerably more targets than MACS in control iPSC. In all cases, the list of target genes identified by MACS was included in the more abundant one obtained using RSEG.

To validate targets identified by RSEG, we applied MACS and RSEG to two recently published H3K27me3 datasets established in mESC ([[1](#_ENREF_1)]; GSE23943: GSM590113 vs. GSM590123). We compared the lists of targets obtained using the indicated peak calling methods to those retrieved from a ChIP-seq experiment aimed to identify SUZ12 targets in the same cells (GSM590121). The latter analysis was performed using the MACS method (with default settings). While the majority of genes identified by both MACS and RSEG overlapped with SUZ12 targets, only a small proportion of genes identified uniquely by RSEG (or through a less stringent MACS analysis) bound SUZ12. Since this observation was at odds with the available biological knowledge, we chose to proceed with the more stringent MACS analysis for the global identification of H3K27me3 targets in iPSC clones.

In contrast, the diffuse pattern of H3K27me2 distribution yielded with a stringent MACS analysis a very limited and poorly reproducible list of target genes. RSEG, instead, identified substantially more targets that overlapped largely between independent iPSC clones of the same genotype. Therefore, we chose to proceed with RSEG for the identification genome wide of H3K27me2 targets.

**Purification of core histones and sample preparation prior MS analysis**

Two representative *Ezh2^+/+^* and *Ezh2^∆SET/∆SET^* IPSC clones were homogenized in lysis buffer (10% Sucrose, 0.5 mM EGTA pH 8.0, 15 mM NaCl, 60 mM KCl, 15 mM HEPES, 0.5% Triton, 0.5 mM PMSF, 1 mM DTT, 5 mM NaF, 5 mM Na_3_VO_4_, 5 mM NaButyrate, Sigma protease inhibitors cocktail); nuclei were fractionated from cytoplasm by centrifugation through 20% sucrose cushions, washed twice in ice-cold PBS and extracted overnight in 0.4 N HCl at 4°C. Solubilized core histones were dialyzed against 100 mM ice-cold CH_3_COOH (6-8 kDa cutoffs). Dialyzed samples were lyophilized and stored at -20°C. Concentration and purity of purified core histones were estimated by Bradford assay and SDS-PAGE analysis on 17% mini gels, stained with Coomassie. For MS analysis, 10 to 20µg of core histones preparations were digested in solution with endoproteinase Arginine-C (Arg-C; Roche) according to the manufacturer’s protocol, overnight at 37°C. Digested peptides were desalted and concentrated using a combination of reverse-phase C18/Carbon “sandwich” system and ion-exchange (SCX) chromatography, on hand-made nano-columns (StageTips) [[2](#_ENREF_2)]: samples loaded on C18/Carbon sandwich and SCX StageTips where eluted with high organic solvent (80% ACN) and NH_4_OH, respectively. Eluted peptides were lyophilized, re-suspended in 0.1% TFA in ddH2O, pooled and subjected to mass spectrometry.

**Mass Spectrometry Analysis (LC-MS/MS)**

Each core histone sample prepared from control and *Ezh2*-mutant cells was analyzed in three technical replicates. Peptide mixtures were separated by nano-LC/MSMS using an Agilent 1100 Series nanoflow LC system (Agilent Technologies), coupled to a 7-Tesla LTQ-FT-ICR-Ultra mass spectrometer (ThermoFisher Scientific, Bremen, Germany). The nanoliter flow LC was operated in one column set-up with a 15 cm analytical column (75 μm inner diameter, 350 μm outer diameter) packed with C18 resin (ReproSil, Pur C18AQ 3 μm, Dr. Maisch, Germany). Solvent A was 0.1% FA and 5% ACN in ddH2O and solvent B was 95% ACN with 0.1% FA. Samples were injected in an aqueous 0.1% TFA solution at a flow rate of 500 nl/min. Peptides were separated with a gradient of 0-40% solvent B over 90 min followed by a gradient of 40-60% for 10 min and 60-80% over 5 min at a flow rate of 250 nl/min. The mass spectrometer was operated in a data-dependent mode to automatically switch between MS and MS/MS acquisition. In the LTQ-FT full scan MS spectra were acquired in a range of *m*/*z* 300 to 1350 by FTICR with resolution *r* = 100,000 at *m*/*z* 400 with a target value of 2,000,000. The five most intense ions were isolated for fragmentation in the linear ion trap using collision-induced dissociation at a target value of 5,000. Singly charged precursor ions were excluded. The nano-electrospray ion source (Proxeon Biosystems, Odense, Denmark) was used with a spray voltage of 2.4 kV. No sheath and auxiliary gasses were used and capillary temperature was set to 180ºC. Collision gas pressure was 1.3 millitorrs and normalized collision energy using wide band activation mode was 35%. Ion selection threshold was 250 counts with an activation q = 0.25. The activation time of 30 ms was applied in MS2 acquisitions.

**MS data analysis and assignment of modification sites using MASCOT and MaxQuant**

Raw data from MS analysis on LTQ-FT Ultra were converted to mgf files using Raw2MSM software [[3](#_ENREF_3)]. MS/MS spectra were first searched by Mascot Daemon (version 2.2.2, Matrix Science) against the IPI mouse database (version 3.63) (56073 sequences; 25214299 residues). MS mass tolerance was initially set to 7 ppm and MS/MS mass tolerance was set to 0.5 Da. Variable modifications included mono- and di-methylation on Lysine and Arginine residues, tri-methylation on Lysines, oxidation on Methionine and acetylation on proteins N-termini. In order to exclude from Mascot results the low-confidence peptide identifications, the following criteria were used: peptides with ion score lower than 15 and more than 5 putative PTMs were removed; redundant peptides with same ID were excluded, by selecting the peptide with the highest Mascot score. Filtered data were then validated by manual inspection, using Qual Browser version 2.0.7 (ThermoFisher Scientific). Sequence assignments produced by Mascot were further processed with MaxQuant software, version 1.0.13.13 [[4](#_ENREF_4)] in order to employ the posttranslational modification (PTM) score analysis, as described previously [[5](#_ENREF_5)]. Briefly, peak- lists generated from the “quant” module were searched using Mascot parameters as above, but with the difference that the IPI mouse database was combined with a list of 175 common contaminants, and concatenated with the reversed versions of all sequences (the so-called “Decoy database”, [[6](#_ENREF_6)]. Up to two missed cleavages were allowed for Arg-C digestion. The “identify” module in MaxQuant was employed to filter identifications at 1% false discovery rate (FDR) at three levels namely: site, peptide, and protein. In each peptide the modification sites was assigned and the quality of this assignment was measured using the “localization probability” calculated by MaxQuant [[7](#_ENREF_7)]. While Mascot and PTM scores evaluate the quality of identification of modified peptides, localization probability measures how confidently a modification can be assigned to a specific site, basing on the information available in the fragmentation spectrum. This approach has been used extensively for phosphopeptides analysis and recently adopted also for other modifications analysis [[8](#_ENREF_8)].

**Supplementary references**

1. Marks H, Kalkan T, Menafra R, Denissov S, Jones K, et al. (2012) The transcriptional and epigenomic foundations of ground state pluripotency. Cell 149: 590-604.

2. Rappsilber J, Mann M, Ishihama Y (2007) Protocol for micro-purification, enrichment, pre-fractionation and storage of peptides for proteomics using StageTips. Nat Protoc 2: 1896-1906.

3. Olsen JV, de Godoy LM, Li G, Macek B, Mortensen P, et al. (2005) Parts per million mass accuracy on an Orbitrap mass spectrometer via lock mass injection into a C-trap. Mol Cell Proteomics 4: 2010-2021.

4. Cox J, Mann M (2008) MaxQuant enables high peptide identification rates, individualized p.p.b.-range mass accuracies and proteome-wide protein quantification. Nat Biotechnol 26: 1367-1372.

5. Olsen JV, Blagoev B, Gnad F, Macek B, Kumar C, et al. (2006) Global, in vivo, and site-specific phosphorylation dynamics in signaling networks. Cell 127: 635-648.

6. Kall L, Storey JD, MacCoss MJ, Noble WS (2008) Assigning significance to peptides identified by tandem mass spectrometry using decoy databases. J Proteome Res 7: 29-34.

7. Nagaraj N, D'Souza RC, Cox J, Olsen JV, Mann M (2010) Feasibility of large-scale phosphoproteomics with higher energy collisional dissociation fragmentation. J Proteome Res 9: 6786-6794.

8. Zielinska DF, Gnad F, Wisniewski JR, Mann M (2010) Precision mapping of an in vivo N-glycoproteome reveals rigid topological and sequence constraints. Cell 141: 897-907.
